# Comparative Proteomics Analyses of Two Races of *Fusarium oxysporum* f. sp. *conglutinans* that Differ in Pathogenicity

**DOI:** 10.1038/srep13663

**Published:** 2015-09-03

**Authors:** Erfeng Li, Jian Ling, Gang Wang, Jiling Xiao, Yuhong Yang, Zhenchuan Mao, Xuchu Wang, Bingyan Xie

**Affiliations:** 1 The Institute of Vegetables and Flowers, Chinese Academy of Agricultural Sciences, Beijing 100081, China; 2 The Institute of Tropical Biosciences and Biotechnology, Chinese Academy of Tropical Agricultural Sciences, Haikou Hainan 571101, China

## Abstract

*Fusarium oxysporum* is a soil-inhabiting fungus that induces vascular wilt and root rot in a variety of plants. *F. oxysporum* f. sp. *conglutinans* (Foc), which comprises two races, can cause wilt disease in cabbage. Compared with race 1 (52557^−TM^, R1), race 2 (58385^−TM^, R2) exhibits much stronger pathogenicity. Here, we provide the first proteome reference maps for Foc mycelium and conidia and identify 145 proteins with different abundances among the two races. Of these proteins, most of the high-abundance proteins in the R2 mycelium and conidia are involved in carbohydrate, amino acid and ion metabolism, which indicates that these proteins may play important roles in isolate R2’s stronger pathogenicity. The expression levels of 20 typical genes demonstrate similarly altered patterns compared to the proteomic analysis. The protein glucanosyltransferase, which is involved in carbohydrate metabolism, was selected for research. We knocked out the corresponding gene (*gas1*) and found that Foc-∆gas1 significantly reduced growth rate and virulence compared with wild type isolates. These results deepened our understanding of the proteins related to *F. oxysporum* pathogenicity in cabbage *Fusarium* wilt and provided new opportunities to control this disease.

The plant pathogen *Fusarium oxysporum* is a soil-borne fungus. It invades roots and can induce vascular wilt and root rot in host plants by colonizing xylem tissue^1^,^2^. More than 120 different *formae speciales* were identified based on host species specificity^3^. Pathogenicity variation of *F. oxysporum* in crucifers have been classified into three *formae speciales*: *conglutinans*, *raphani* and *mathioli*^4^. *F. oxysporum* f. sp. *conglutinans* (Foc) induces vascular disease in cabbage. Cabbage is one of the most important vegetables and is widely grown in Northern China. In recent years, its production has sustained heavy losses from *Fusarium* wilt due to *F. oxysporum*^5^. Cabbage *Fusarium* wilt originated in the United States, and it was first reported in China in 2001^6^,^7^. The races were named due to the different pathogenicity in a *formae specials*^8^. Isolates with a different specificity toward cabbage genotypes were referred to as race1 (R1, 52557^−TM^) and race2 (R2, 58385^−TM^). These two races were identified based on their reactions with different cabbage genotypes. R1 is a relatively weak pathogenic isolate; however, R2 is a strong pathogenic isolate that can overcome monogenic, type A resistance in cabbage^9^. Why are the pathogenicity and growth rate different among these two races? Unfortunately, the answers have not yet been established.

Proteomics is a powerful tool that can provide global information for a large number of proteins expressed in a particular biological state by supplying an accurate analysis of cellular protein status and changes during a specific state^10^,^11^,^12^. In this study, we aimed to clarify the main pathways in isolate R2, which features the stronger pathogenicity, using comparative proteomic analysis. The wood-degrading fungi *Phaenaerochaete chrysosporium* and *Lentinula edodes* were two of the earliest intracellular filamentous fungi studied using proteomics^13^. Grinyer *et al*. were first to use mass spectrometry (MS) to identify proteins in filamentous fungi^14^. Proteomic studies on filamentous fungi have been performed extensively in recent years with an increasing number of species that have been sequenced and annotated, but few proteomic datasets are available^10^. The *Botrytis cinerea*, *Sclerotinia sclerotiorum* and *Verticillium albo-atrum* mycelium proteomes have been studied^15^,^16^,^17^,^18^. The studies were an attempt to identify and analyse the proteins with different levels of abundance among strains with different pathogenicity. Proteomic studies of conidia have also been conducted on *Uromyces appendiculatus*, *Blumeria graminis*, *Nomuraea rileyi*, *Trichophyton rubrum* and *Metarhizium acridum*^11^,^19^,^20^,^21^,^22^. The proteins identified in different species’ conidia exhibited metabolic protein abundance levels that are similar to the metabolically active fungal mycelium. Despite the extensive proteomics studies on filamentous fungi, few proteomic studies have been performed on *F. oxysporum*.

For filamentous fungi, mycelium are the structures formed during vegetative growth to provide nutrition for survival; conidia are specialized structures that are frequently responsible for dispersal and environmental persistence^23^. Conidia are also the asexual propagation units of many fungal plant pathogens and play important roles in fungal propagation and host infection^2^. Therefore, in this work, we sought answers through a comparative proteomics study of the mycelium and conidia of two races. Discriminating the factors between races that exhibit different pathogenicity towards a specific plant is an essential step to discerning the pathogenic proteins and providing effective disease control measures against *F. oxysporum* infection. The main objectives of this study were to provide high resolution proteome reference maps for Foc mycelium and conidia and to perform a comparative proteomic analysis between R1 and R2. We identified the proteins that exhibited different abundances in the mycelium and conidia among the two races. These studies are crucial to understanding the biological and molecular basis of these specialized structures in this fungal pathogen’s life cycle. Furthermore, the studies will aid in identifying the proteins with different abundances that are related to R2’s stronger pathogenicity. Further, such information may help clarify the pathogenicity mechanism underlying this important pathogen, which will aid in disease control.

## Results

### Comparison of growth and pathogenicity between R1 and R2

**Figure 1:**
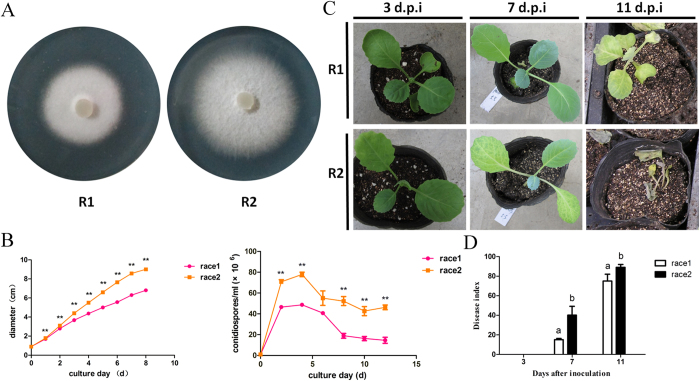
Comparison of growth and pathogenicity between R1 and R2: (A) the growth of *Fusarium oxysporum* f. sp *conglutinans* R1 and R2 after a 5-day culture on PDA media; (B) the growth rates and conidial concentrations of R1 and R2. Each data point represents the average of three independent biological replicates. The average is shown with the SE of the mean. ** represents the significant differences at *p* < 0.01; (**C**) the disease symptoms induced by R1 and R2 of Foc on Chinese cultivar “Zhong Gan 21”. The cabbage seedlings are shown at 3, 7, 11 days after inoculation; (**D**) the disease index analysis for cabbage seedlings inoculated with R1 and R2 at 3, 7, 11 days after inoculation. Each value of the disease index is the average of three independent biological replicates. Each test on the disease index was performed on more than thirty seedlings. The values followed by different letters were significantly different according to Duncan’s multiple range tests at *p* < 0.01.

We compared the growth rates between R1 and R2 cultured on PDA medium from 1 to 8 days by measuring the isolate diameters (Fig. 1A). As shown in Fig. 1B, R2 grew at a more rapid rate than R1 after 1 day of culture. Both R1 and R2 produced conidia after 24 h. However, R2 produced a significantly more conidia (Fig. 1B) compared with R1.

Disease symptoms ranged from yellowing leaves to vascular discoloration in stems and wilting seedlings, and the symptoms were assessed on days 3, 7, and 11 after inoculation. The cabbage disease symptoms upon inoculation with R1 and R2 are shown in Fig. 1C. Leaf chlorosis was induced by R2 much earlier and with greater severity than the R1 infection. We further used the disease index to evaluate the difference in pathogenicity between R1 and R2. As shown in Fig. 1D, R2 was more aggressive and induced more severe symptoms in the same cabbage seedling genotype compared with R1.

### General description and global results from the proteomic analysis

**Figure 2:**
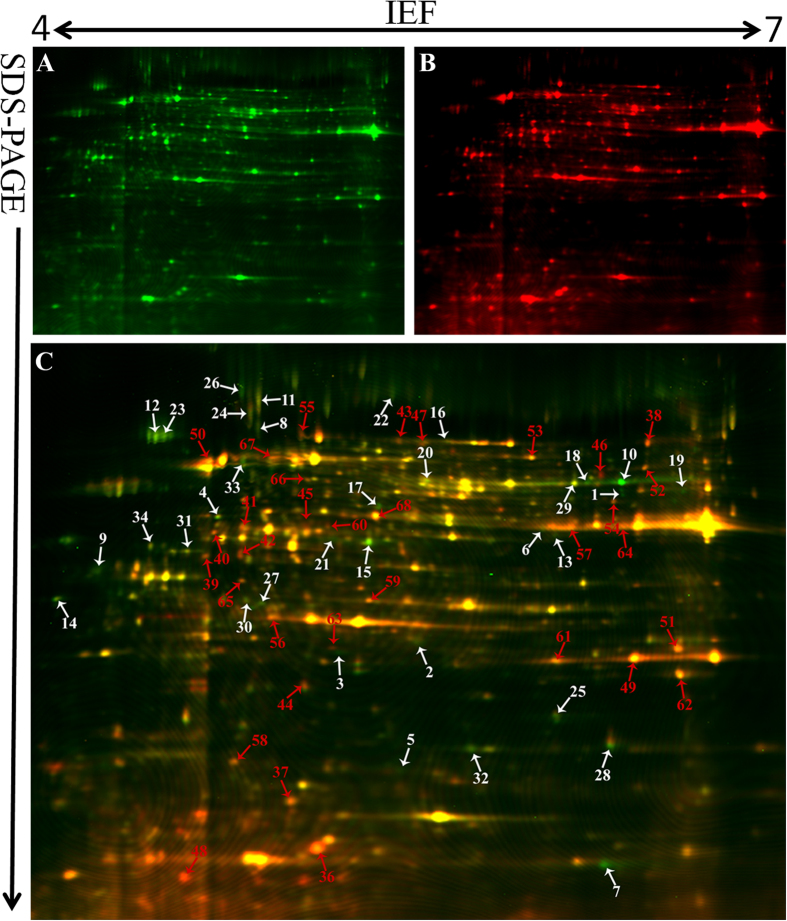
Reference map from 2-D DIGE analysis of *F. oxysporum* f. sp *conglutinans* mycelia proteins. (**A**) Mycelia proteins from R1 were labelled with Cy3 (green); (**B**) mycelia proteins from R2 were labelled with Cy5 (red); (**C**) overlay of the two pictures. The gel was obtained using 24 cm, pH 4–7 IPG strips for IEF and followed by 12.5% SDS-PAGE. The proteins identified with different abundances are indicated by arrows and numbers and are listed in Table 1. The white arrows and numbers represent the proteins identified in R1 mycelium, and red represents the proteins identified in R2 mycelium.

**Figure 3:**
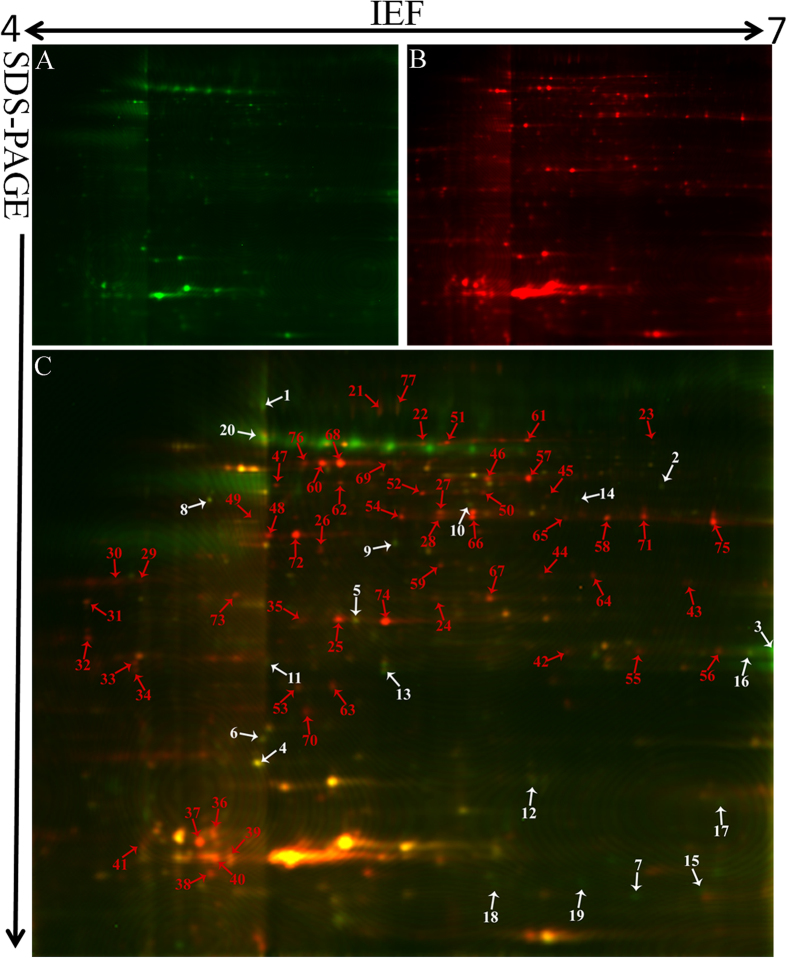
Reference map from 2-D DIGE analysis of *F. oxysporum* f. sp *conglutinans* conidial proteins. (**A**) Conidia proteins from R1 were labelled with Cy3 (green); (**B**) conidia proteins from R2 were labelled with Cy5 (red); (**C**) overlay of the two pictures. The gel was obtained using 24 cm, pH 4–7 IPG strips for IEF and followed by 12.5% SDS-PAGE. The proteins identified with different abundances are indicated by arrows and numbers and are listed in Table 2. The white arrows and numbers represent the proteins identified in R1 conidia, and red represents the proteins identified in R2 conidia.

**Figure 4:**
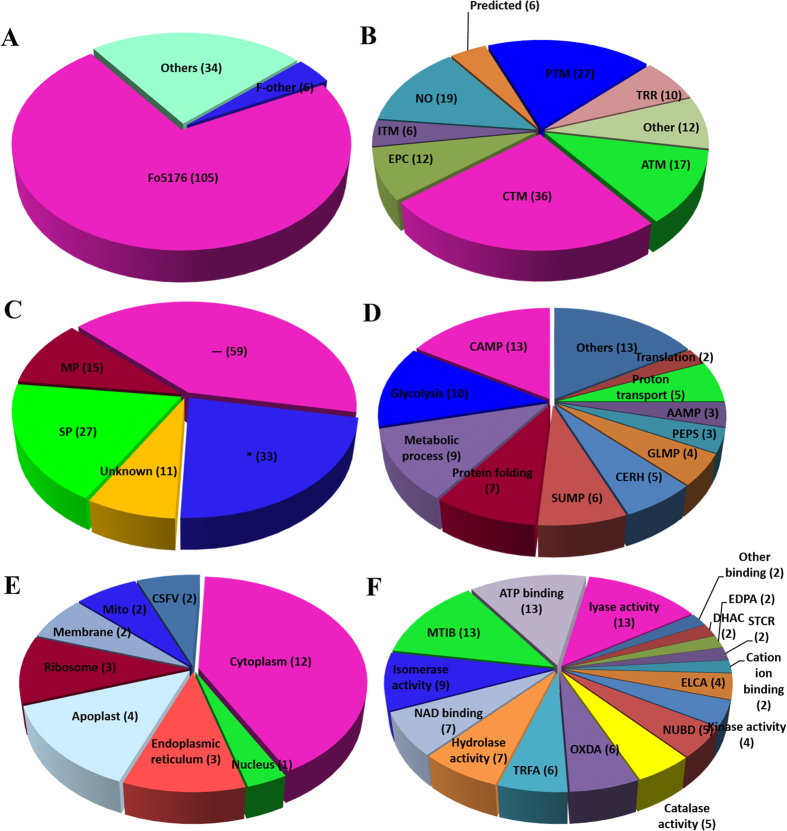
Classification, subcellular location and functional analysis of the identified proteins: (A) the distributions of the 145 proteins identified in different species. The abbreviations for the distributions in different species are: Fo5176, *F. oxysporum* Fo5176; F-other, other *Fusarium* species; and Others, other fungi; (**B**) the proportion of the identified proteins in each category of the COG classification system; (**C**) The subcellular locations of the identified proteins determined using TargetP; the analysis for biological process (**D**), cellular component (**E**) and molecular function (**F**) using Go classification was also performed. The abbreviations for the COG classification system as well as TargetP and GO classifications are provided in detail in Supporting Information 1.

We used DeCyder software for the proteomic analysis, which showed more protein spots in mycelium compared with conidia. Only 207 protein spots exhibited more than a 1.5-fold change that was statistically significant (*p* < 0.05). Furthermore, 145 protein spots were successfully identified by MALDI TOF/TOF MS/MS (Supporting Information 1). The molecular mass of the identified proteins ranged from approximately 4 to 73 kDa. The protein spots identified are indicated by arrows and numbers on the gels (Fig. 2 and Fig. 3). Based on the identification results, 72% (105/145) proteins with different abundances in R1 and R2 were identified in *F. oxysporum* Fo5176. Six proteins were also identified in other *F. oxysporum formae speciales* (Fig. 4A). The protein spots were functionally annotated through searching the UniProt database. The identified proteins were grouped into the main known metabolic pathways based on their putative functions, which were determined using the COG classification system.

Among the proteins, the carbohydrate transport and metabolism-related proteins (CTM, 36) were most abundant, which were followed by posttranslational modification chaperone-related proteins (PTM, 27), amino acid transport and metabolism (ATM, 17), and energy production- and conversion-related proteins (EPC, 12). The remaining proteins featured various functional annotations (34). Nineteen protein spots belong to an unknown function classification (Fig. 4B). The subcellular locations of these proteins were mainly classified as any other location (59 proteins, _), which was followed by 33 of unknown location (*) and 27 in the secretary pathway (SP) (Fig. 4C). Gene ontology (GO) analysis at three different levels (biological process, cellular component, and molecular function) was performed for the 145 proteins with different abundances in R1 and R2. The GO analysis for biological processes revealed that most proteins were involved in carbohydrate metabolic processes (CAMP) and glycolysis (Fig. 4D). The cellular component GO analysis showed that the largest portion of proteins, which comprised 12 proteins, was overrepresented in the cytoplasm (Fig. 4E). Finally, the largest portion of the molecular function GO analysis was involved in metal ion binding (MTIB), ATP binding, and lyase activity (Fig. 4F).

Compared with R1, R2 produced 81 high-abundance, 46 low-abundance, and 18 specific spots. The R2-specific proteins were involved in inorganic ion transport and metabolism. Our research shows that the proteins related to carbohydrate metabolism, amino acid metabolism and inorganic ion transport and metabolism are highly abundant in R2 with stronger pathogenicity. The proteins with different abundances related to carbohydrate metabolism comprised the largest portion of both the mycelium and conidia in R2. The results are schematically depicted and analysed below. Details of the identified proteins are listed in Supporting Information 1.

### Differential proteins between the R1 and R2 mycelium

**Figure 5:**
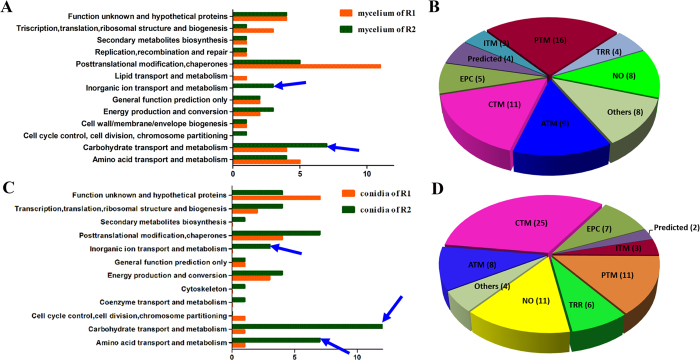
A comparative analysis of the functional categories for the identified proteins in the mycelium and conidia between R1 and R2: the functional categories of identified mycelia proteins (A) and conidial proteins (C) between R1 and R2; the proportion of identified mycelia proteins (B) and conidial proteins (D) in each COG classification category; the blue arrows indicate the crucial functional categories that represent the most abundant proteins in R2.

We found 93 highly reproducible protein spots that exhibited significantly different abundances (≥±1.5-fold) between the mycelium samples of the two races; these spots were further analysed using a PMF or an MS/MS analysis. A total of 68 protein spots, 35 from R1 mycelium and 33 from R2 mycelium, were identified and are indicated in Fig. 2. Among the protein spots, 60 proteins were predicted to provide known biological functions (Table 1). Based on their putative functions, the protein spots were classified into 13 groups using the biological process annotation program COG (Fig. 5A). Among the proteins, posttranslational modification chaperones (PTM) and carbohydrate transport and metabolism (CTM)-related proteins were most abundant, which were followed by amino acid metabolism (ATM)-related proteins (Fig. 5B). Eight protein spots were identified as hypothetical proteins.

Carbohydrate metabolism proteins were identified in both R1 (11%) and R2 (21%) and exhibited especially high levels in R2 (Fig. 5A). This group includes proteins such as glyceraldehyde-3-phosphate dehydrogenase (spot 64), transketolase (spot 41), glucose-epimerase (spot 40), glucose-isomerase (spot 53), triose-phosphate isomerase (spot 56), enolase (spot 67), and adenosine kinase (spot 68). Glyceraldehyde-3-phosphate dehydrogenase, glucose-epimerase, glucose-isomerase, and adenosine kinase are involved in glycolysis, and transketolase is involved in the pentose phosphate pathway^24^. β-1, 3-glucanosyltransferase was also identified. This protein is implicated in fungal cell wall biosynthesis and plays an essential role in *F. oxysporum* pathogenesis in tomato^25^.

In this study, three proteins (spots 49, 61, and 62) that are related to inorganic ion transport and metabolism were identified as superoxide dismutase in R2 (Table 1). Superoxide dismutase can ameliorate hostile environment created by host reactive oxygen species (ROS).

As shown in Table 1, 16 spots were identified as posttranslational modification proteins or chaperones. Eleven such proteins in R1 were positively identified (spots 8, 9, 11, 12, 14, 22, 23, 26, 27, 31 and 34). In R2, 5 proteins (spots 39, 55, 58, 59 and 63) were positively identified. Spots 39, 55 and 58 were identified as heat shock proteins (HSP) in the R2 mycelium. HSP proteins are highly conserved and present in each cell of each organism. Molecular chaperones act in protein biogenesis and regulation under normal growth conditions; they play crucial roles in the cellular response to stress^26^. Furthermore, protein spots 59 and 63 in R2 were identified as proteasome subunits. In eukaryotes, the proteasome is an essential component of the ATP-dependent proteolytic pathway. For example, the 20S proteasome plays multiple roles in peptidase activities^27^. Abundant proteins that are related to posttranslational modification and chaperones were identified, which indicates an elaborate system of chaperones and folding catalysts to ensure that proteins participate in the proper reactions and that folding proteins do not participate in improper interactions in Foc. In our research, we commonly observe different protein spots that are identified as identical proteins through functional annotation. Previous studies explain that this might result from either post-translational modifications of the same gene product or from sequence-related isoforms encoded by distinct genes^28^. In this study, these observations might also be due to post-translational modification-related protein abundance in Foc.

In our study, proteins related to energy metabolism were abundant in both R1 and R2 to maintain normal growth, development, and differentiation. Additional proteins, such as glucose-epimerase (spot 54), aldo/keto reductase (spot 57), ketoreductases (spot 44), and cryptochrome-2 (spot 48), were also highly abundant in R2.

Spots 5, 6, 13 and 25 in R1 were identified as putative proteins from *F. oxysporum*, *Coccidioides immitis*, and *Gibberella zeae*, respectively. In R2, spots 36, 42, 51, and 65 were identified as hypothetical proteins from *F. oxysporum* Fo5176.

### Proteins with different abundances between the R1 and R2 conidia

The DIGE analysis revealed that 114 proteins exhibit highly reproducible and significantly different abundances (≥±1.5-fold) when conidia samples from the two races are compared. The proteins were subjected to an MS analysis. A total of 77 protein spots from R1 (20) and R2 (57) conidia were successfully identified using a combination of PMF and MS/MS fragmentation (Fig. 3). Among these protein spots, 66 featured known biological functions (Table 2). The conidia proteins identified are involved in many different biological processes and were divided into 12 groups (Fig. 5C). Among these proteins, carbohydrate transport and metabolism (CTM)-related proteins were most abundant, which are followed by posttranslational modification and chaperone-related proteins as well as amino acid transport and metabolism proteins (Fig. 5D).

The proteins related to carbohydrate transport and metabolism were at the highest percentage (42%) in the R2 conidia. Triose-phosphate isomerase (spots 25, 35 and 74), fructose aldolase (spots 27, 28 and 66), glyceraldehyde-3-phosphate dehydrogenase (spots 65, 71 and 75), phosphoglycerate kinase (spots 46 and 57), adenosine kinase (spot 54), and transaldolase (spot 72) were identified in R2. Spots 29, 30, 32, 33, 34, 37 and 41 were identified as glycosidase, which was highly abundant and had seven isoforms in R2 conidia. Spots 60, 68, 73, and 76 were identified as enolase in R2. Protein spot 20 was identified as β-1, 3-glucanosyltransferase, which was also detected in the mycelium.

The proteins related to inorganic ion transport and metabolism were also only identified in R2 conidia. Spots 42, 55 and 56 were identified as superoxide dismutase, which was also only detected in R2 mycelium. Superoxide dismutase can degrade ROS created by host plants and allow further infection and colonization by a pathogen^29^. We speculated that this protein might play an important role in R2’s stronger pathogenicity.

Seven high-abundance proteins related to amino acid transport and metabolism were detected in R2. Spots 22, 51, 61, and 69 were identified as pyruvate decarboxylase. Spots 45, 59, and 62 were identified as isocitrate dehydrogenase, spermidine synthase, and saccharopine dehydrogenase, respectively. Our results indicate that more proteins related to protein metabolism were present in the R2 conidia compared with R1.

Four proteins were identified as molecular chaperones in the R2 conidia, including Hsp90 co-chaperone p23 (spot 31), small heat-shock proteins (spot 44, 64) and Hsp70-like protein (spot 77). P23 is a Hsp90 component that binds and stabilizes the ATP-bound dimeric form of Hsp90. Ruth Matthews and James Burnie suggested that fungal Hsp90 played a key pathogenic role in systemic infection^30^. A powerful and effective strategy for fungal infectious disease is to harness Hsp90 function^31^. In Foc conidia, the HSP protein abundance may enhance conidial tolerance to environmental stress and participate in virulence. Peptidyl-prolyl isomerase was also identified in spots 38, 39 and 40 in R2; peptidyl-prolyl isomerase plays a role in many cellular processes, such as protein folding, pre-mRNA splicing, and cellular signalling^32^.

In the R1 conidia, 35% of the identified proteins were hypothetical or proteins with unknown function from *F. oxysporum*. Spots 7, 15, 18, and 19 were identified as Fo5176-SIX1, which was grouped with the hypothetical proteins and exhibited high abundance in R1. In the R2 conidia, spots 24, 36, 63, and 70 were identified as hypothetical proteins.

### Comparing the gene expression patterns for the 20 typical proteins in the R1 and R2 mycelium and conidia

**Figure 6:**
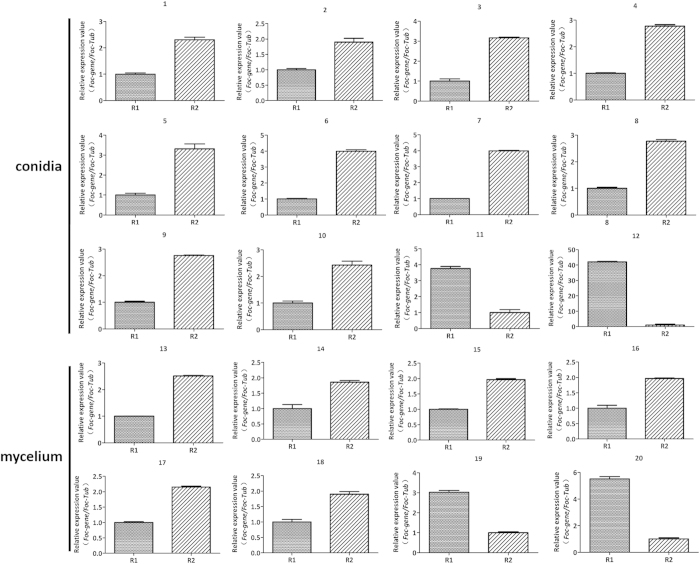
Comparison of the gene expression patterns for the 20 typical proteins in R1 and R2. The relative expressions at the mRNA level of the mycelium and conidia proteins with different abundance in R1 and R2. *β-tubulin* was used as reference gene. Each rectangle denotes the average of four biological replicates with standard errors.

To further evaluate the differences in spot abundance for the proteins identified in the proteomic analysis, we used qRT-PCR analysis for 20 typical proteins, including 8 proteins from mycelium and 12 from conidia, which were mainly from R2. In general, the qRT-PCR analysis of the expression levels of the typical genes demonstrated that the patterns of change were similar to the protein spot abundance changes in our proteomic analysis (Fig. 6). The highly abundant proteins in R2 also exhibited higher mRNA expression levels in R2 than that in R1.

### *Gas1* gene deletion in Foc and the functional analysis of the mutant Foc-∆gas1

**Figure 7:**
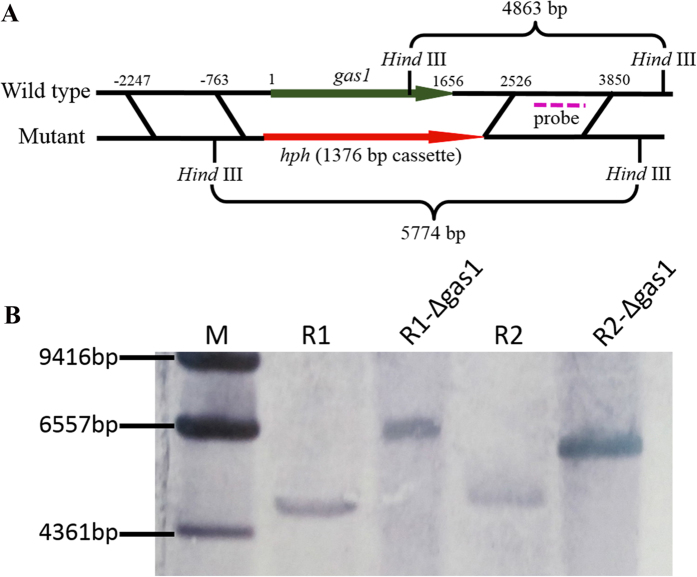
Construction and verification of the Foc-∆gas1 deletion mutants. (**A**) Foc-gas1 gene deletion and replacement with an intact selectable marker gene (*hph*) through homologous recombination; (**B**) southern hybridization analysis for wild type isolates and Foc-∆gas1 mutants. The wild type isolates (Foc) and deletion mutants (Foc-∆gas1, with a hygromycin-resistance cassette) were digested with *Hind*III. A fragment amplified from downstream of the target gene was used as a probe.

To further evaluate the speculations based on the proteomic analyses, one protein that was identified as glucanosyltransferase, which is involved in carbohydrate transport and metabolism, was selected for further research. We deleted the corresponding gene (*gas1*) and discussed the roles of this protein in Foc. *Gas1* was deleted and replaced with an intact selectable marker gene (*hph*) by homologous recombination (Fig. 7A). The split replacements for the selectable marker gene, which comprised a 1484 bp region upstream and a 1324 bp region downstream, were amplified as shown in Supporting Information 2 Figure S1. Southern hybridization analyses show that the Foc-∆gas1 deletion mutants lacked the target gene and instead featured the intact selectable marker gene in contrast to the wild type isolates (Fig. 7B).

**Figure 8:**
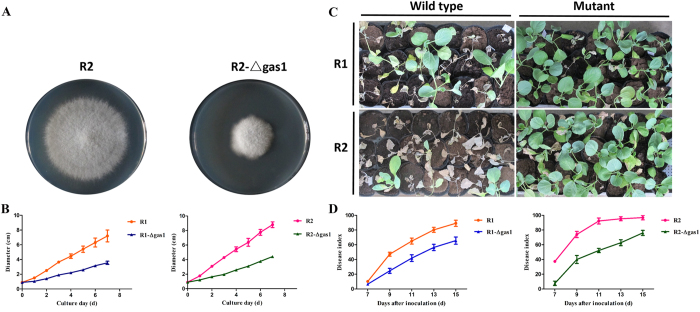
Function analysis of *gas1* in *F. oxysporum* f. sp. *conglutinans*. (**A**) The colony morphology of R2 and Foc-∆gas1 after a 7-day culture on PDA; (**B**) the growth rates of wild type isolates (R1 and R2) and the mutations (Foc-∆gas1). Each data point represents the average of three independent biological replicates. The average of three replicates is shown with standard errors of the mean; (**C**) disease symptoms induced by wild type isolates and mutations (Foc-∆gas1) in Chinese cultivar “Zhong Gan 21” at 13 days after inoculation; (**D**) The disease index analysis of cabbage seedlings inoculated with wild type isolates (R1 and R2) and the mutations (Foc-∆gas1) at 7, 9, 11, 13 and 15 days after inoculation. Each value for the disease index is an average of three independent biological replicates. Each test for the disease index was performed using more than thirty seedlings.

To explore the roles of glucanosyltransferase in Foc, we compared the growth rates of wild type isolates and the mutations. The deletion mutant Foc-∆gas1 significantly reduced the growth rate and exhibited restricted colony growth in the two races (Fig. 8A). When cultured on PDA medium for 7 days, the R2 growth rates were 2.25-fold greater than for the mutations (Fig. 8B). The wild type isolate and mutant (Foc-∆gas1) growth rates differed significantly as determined by a variance analysis. Therefore, we conclude that this protein plays crucial roles in Foc growth; it is related to cell wall biosynthesis and morphogenesis. We compared the disease symptoms of cabbage seedlings inoculated with wild type isolates and mutants to examine their roles during infection. The Foc-∆gas1-inoculated cabbage disease symptoms were significantly reduced. As shown in Fig. 8C, at 13 dpi, the severe necrotic and wilt symptoms were visualized in the cabbage inoculated with wild type isolates, but the seedlings inoculated with Foc-∆gas1 only showed netted yellowing of the leaves. For example, at 13 dpi, the disease index of cabbage infected with wild type R2 was 95, while the disease index for the cabbage infected with Foc-∆gas1 was 63 (Fig. 8D). The wild type isolates of the two races and the mutations (Foc-∆gas1) exhibited significantly different virulence, which was determined using a variance analysis. The data indicate that glucanosyltransferase may be involved in virulence, which could delay the disease development, but not prevent the disease.

## Discussion

### Proteins involved in carbohydrate transport and metabolism may be important for R2’s stronger pathogenicity

High quantities of ATPase are necessary to provide energy for growth, development, differentiation, and sporulation. ATPase is mainly produced through carbohydrate metabolism^33^. In our study, we indicate that the proteins related to carbohydrate metabolism, such as enolase, glyceraldehyde-3-phosphate dehydrogenase, phosphate isomerase, and glycosidase, exhibited high levels in mycelium of R2 (21%) and exhibited the highest percentage (42%) in R2 conidia. Although a direct role for the proteins in R2 is unclear, the greater abundance of proteins related to carbohydrate metabolism in R2 than in R1 suggests that the proteins play a crucial role in the more rapid growth and stronger pathogenicity of R2.

The enolase abundance was high in the R2 mycelium and conidia. Enolase is a multifunctional protein that participates in a variety of cellular activities; for example, it acts as an HSP, binds cytoskeletal and chromatin structures to modulate transcription, and plays a crucial role in pathophysiological processes^34^. Similar results were observed in research on *Fusarium oxysporum* f. sp. *cubense*. Enolase exhibited high abundance in R4, which shows stronger virulence than R1^35^. Based on our results, the enolase abundance was also high in R2 conidia.

Glyceraldehyde-3-phosphate dehydrogenase was highly abundant in the two races, especially R2. This protein participates in the glycolytic cycle; it also influences many other cellular processes and acts as a virulence factor in *B. cinerea*^16^. In *Paracoccidioides brasiliensis*, glyceraldehyde-3-phosphate dehydrogenase is a cell surface protein involved in fungal adhesion to extracellular matrix proteins and interaction with cells^36^.

In R2, triose-phosphate isomerase is highly abundant; this protein was also identified in *Blumeria graminis* f. sp. *hordei* conidia^20^. Triose-phosphate isomerase was studied in *Paracoccidioides brasiliensis*. It was required for the interaction between *P. brasiliensis* and extracellular matrix molecules^37^. This protein might play an important role in fungi adherence and host cell invasion.

Glycosidase was highly abundant in R2 conidia and featured seven isoforms. Pegg and Young reported that glycosidase was related to fungal colonization during tomato infection by *Verticillium albo-atrum*^38^. Its roles in Foc were not confirmed. Perhaps this enzyme plays essential roles in Foc.

In addition to the aforementioned proteins with different abundances, other highly abundant proteins related to carbohydrate metabolism were also identified in R2. These proteins were more abundant in R2 than R1, which is consistent with R2’s more rapid growth rate compared with R1 because the fast-growing R2 mycelium requires more energy than R1. The growth rate and quantity of conidia play important roles in pathogen infection and colonization^39^,^40^,^41^,^42^.

The protein glucanosyltransferase is involved in carbohydrate transport and metabolism, and the corresponding gene was deleted to research its roles in Foc. The deletion mutant Foc-∆gas1 exhibited significantly lower growth rates. Moreover, virulence in cabbage was significantly lower compared with wild type isolates. The results are consistent with speculations based on the proteomic analyses, which suggest that the proteins involved in carbohydrate transport and metabolism are important for Foc.

### Proteins involved in amino acid transport and metabolism may play important roles in R2 conidia

In our results, the abundance of proteins related to amino acid metabolism was high in both the R1 and R2 mycelium, and these enzymes might be required for normal mycelia growth and basic metabolism. However, the enzymes were more abundant in R2 conidia compared with R1. These data demonstrate that the R2 conidia might be active and may participate in a variety of metabolic activities, including germination, adsorption, and colonization.

### Proteins related to inorganic ion transport and metabolism may aid in R2’s stronger pathogenicity

To our knowledge, an early plant response following pathogen recognition is generation of ROS as a defence mechanism^43^. ROS act not only as protectants against pathogen infection but also as signals to activate additional plant defence reactions, including a hypersensitive response in infected cells^44^,^45^. In addition, ROS production inhibits spore germination in many fungal pathogens^46^. Therefore, pathogenic fungi must degrade ROS to overcome host defences and further infect as well as colonize the host. In this study, the proteins related to inorganic ion transport and metabolism (ITM) were only identified in R2. Superoxide dismutase was highly abundant in the R2 mycelium and conidia; this enzyme can change a hostile environment to overcome plant defences through degrading ROS at the early infection stage. More rapid ROS degradation may slow the plant defence response and provide sufficient time for the fungus to escape to adjacent vessels^18^. Studies on *Neurospora crassa* have also shown that conidial longevity is positively correlated with superoxide dismutase^29^,^47^, which may yield easier and faster infection and colonization. The high abundance of this protein in R2 may explain the different infection rates between R1 and R2 at the early stage of interaction with the same genotype cabbage.

## Conclusion

Large economic losses for cabbage are caused by the pathogenic fungus *F. oxysporum* f. sp. *conglutinans*; however, little is known regarding the fungus at the protein level. The powerful tools 2-D DIGE and MS are used to separate and identify proteins. Careful systematic and statistical analyses combined with these tools reliably revealed the protein-level differences between Foc two races. This approach is useful for discerning the proteins that are isolate-specific and exhibited different levels of abundance at two different development stages of Foc. In the present study, we report the first proteomic map of Foc and provide a comparative analysis overview on the conidia and mycelium between two races of this pathogen using a proteomic strategy.

In our study, most proteins were detected in the mycelium, which was followed by the conidia; the greatest differences in abundance among the two races were detected in the conidia protein profiles. Host infection and colonization begins from conidia adhesion and germination. We speculate that the proteins with different abundances in the conidia explain the different infection rates and pathogenicity between two races. Identifying the 145 different proteins between the two races and functional analyses for the 90 proteins from the strong pathogenic R2 revealed that the proteins involved in carbohydrate metabolism, amino acid metabolism and ion metabolism might play crucial roles in *Fusarium* cabbage wilt. To confirm our speculations, the protein glucanosyltransferase, which is involved in carbohydrate metabolism, was used to research the role of carbohydrate metabolism in Foc. The results show that this protein is implicated in Foc growth and virulence. Furthermore, among the identified proteins, several enzymes, such as glycosidase and superoxide dismutase, might be important for Foc. Although the functional experiments of these proteins were not performed in this work, these results deepen our understanding of the proteins related to *F. oxysporum* pathogenicity in cabbage *Fusarium* wilt. Further, these results are important for providing new insights into *F. oxysporun*-induced cabbage wilt and new opportunities for controlling this disease.

Fungal pathogenicity requires the coordinated regulation of multiple genes and proteins; thus, future work should involve identifying the remaining proteins and confirming the other potential candidate pathogenic proteins in each isolate. In our results, a common observation included different protein spots that were identified as the same protein. Alternative protein forms may result from post-translational modifications and sequence-related isoforms encoded by distinct paralogue genes. An in-depth analysis of these proteins and isoforms is necessary to better understand their roles in *F. oxysporum*. In this research, we show that different spots yielded the same protein identities. Further, we will investigate the effects of these proteins and their isoforms to expand our understanding of functional complexity in the cell; such data may key to understanding the discrepancy between the number of genes and functional complexity.

## Methods

### Preparation for *F. oxysporum* f. sp. *conglutinans* mycelium and conidia

The two Foc races, R1 and R2, were obtained from the Beijing Academy of Agriculture and Forestry Sciences and selected for use in this study. To prepare for mycelium and conidia, both isolates were separated from single spores and grown at 28 °C on potato dextrose agar (PDA) medium. When the colonies reached 3–4 cm in diameter, they were transferred into 300 mL of autoclaved potato dextrose broth (PDB) and cultured at 28 °C on a shaker at 150 rpm for 72 h. Next, the mycelium were filtered through four layers of sterilized lens papers. The conidia were separated from the conidial suspensions by centrifugation at 5000 × *g* for 15 min. Portions of the conidia were re-incubated under the same conditions, except that the culture time was 48 h to obtain the mycelium. Next, the mycelium were filtered and washed with sterile distilled water three times. The conidia were collected for virulence, protein and RNA extraction. The mycelium were used for protein and RNA extraction. For each sample, three independent biological replicate experiments were performed.

### Pathogenicity experiments

#### Plant material

Cabbage plants of the Chinese cultivar “Zhong Gan 21” were grown in plastic pots filled with an autoclaved mixture composed of vermiculite and turf (1:1, v/v). The pots were maintained in the greenhouse at approximately 25 °C for a 16 h photoperiod until the 2–3 true leaf stage.

#### Inoculum preparation and inoculation procedure

Conidial isolate suspensions for R1 and R2 were used for inoculation. The preparation for the inoculum is described above; the concentration was adjusted to 1 × 10^6^ conidia/mL. Seedlings from 2–3 true leaves were inoculated by the root-dip method^48^. The roots of each seedling were gently washed in tap water and dipped in the conidial suspension for 15 min^5^. In addition, the cabbage seedlings were dipped in sterilized distilled water for the non-inoculated controls. The inoculated and control seedlings were maintained in a greenhouse under the same conditions as mentioned above. The seedlings were placed in a random complete block design. For each race, three independent biological replicate experiments were performed, and thirty seedlings were inoculated in each replicate.

#### Disease assessment and statistical analysis

The disease symptoms were assessed after inoculation. The disease severity was divided into five levels based on pre-established rating scales^5^. The scoring standard for cabbage susceptibility to Foc was evaluated through statistical analyses using an index = 0–5, where 0 = no evidence of disease, and 5 = death. The statistical analyses were performed using Duncan’s multiple range tests and SAS software. The statistical results are shown as the mean ± SE (standard error).

### Proteomic analysis of R1 and R2

#### Protein extraction and quantification

The total proteins were extracted from the mycelium and conidia of two races using the BPP protocol^49^. This method combines ammonium sulphate saturated-methanol and Borax/PVPP/Phe to isolate high-quality proteins with good yields. Briefly, five grams of the mycelium and conidia from R1 and R2 were used separately for protein extraction. The protein concentrations were quantified using a spectrophotometer and the Bradford method. Bovine serum albumin (BSA) was used as the standard^50^. By calculating the corresponding concentration, approximately 50 μg of each protein sample was transferred to a new PCR tubes for DIGE, and the PH was maintained at 8.5 using 2 M Tris. Approximately 1300 μg protein sample was used for the 2-DE experiments. The prepared samples were stored at −20 °C.

#### Cy-Dye labelling and 2-D DIGE experimental design

The proteins were labelled using Cy-Dye DIGE Fluors (GE Healthcare) in accordance with the product booklet. The dry Cy-Dye was reconstituted in high-quality anhydrous dimethylformamide (DMF) to prepare a working solution of 0.4 mM. The working solution should be used immediately. Approximately 1 μl working solution was used to label 50 μg protein from the sample. Each sample was labelled with different dyes, Cy2, Cy3 and Cy5. The Cy2 dye was used to label an internal standard, which was prepared by mixing four samples from R1 and R2 mycelium and conidia equally. The Cy3 and Cy5 dyes were used to label the two protein samples from R1 and R2 that were compared. The labelling mixtures were incubated on ice in the dark for 30 min. We added 1 μl of 10 mM lysine to stop the reaction. The reaction was incubated for 10 min on ice in the dark. After the protein samples were labelled, we added lysis buffer containing urea, thiourea, CHAPS and DTT for a volume of 450 μl; we added 5 μl IPG 4–7 buffer (GE Healthcare, Amersham Biosciences) to the mixtures prior to IEF. The samples labelled with three different dyes were performed on a single 2-D gel. The experimental design in Supporting Information 2 Table S1 was used. Three biological replicate experiments were performed.

#### 2-DE (IEF/SDS-PAGE) and gel analysis

The 24 cm, pH 4–7 linear gradient IPG strips (Immobiline DryStrip, GE Healthcare Bio-Sciences AB, Uppsala, Sweden) were rehydrated with the mixtures on a rehydration tray. Next, the strips were covered with fluid oil (Dry Strip Fluid, GE Healthcare) and incubated horizontally for 18 h at 22 °C. IEF was performed using a Hoffer IEF100 at 20 °C for focusing. Thereafter, each IPG-focused strip was equilibrated twice in equilibration buffer (50 mM Tris pH 8.8, 6 M urea, 30% glycerol, 2% SDS, and 0.002% bromophenol blue) containing 1% DTT for 8 min with gentle shaking and subsequently equilibrated twice for 8 min in 5 mL equilibration buffer containing 4% iodoacetamide. The equilibrated strips were transferred to 12.5% sodium dodecyl sulphate polyacrylamide gel electrophoresis (SDS-PAGE) to separate the proteins in the second dimension (Ettan DALT six, GE Healthcare). Three biological replicates were performed for each sample to ensure reproducibility.

The gels were scanned by a Typhoon^TM^ Trio imager (GE Healthcare, Amersham Biosciences). The labelled proteins were visualized in the fluorescence mode and analysed as well as compared using the DeCyder 2D Software v7.0 (GE Healthcare). The Cy2 gel images were defined as standards; each individual Cy3 gel image was assigned as control, and the Cy5 images were assigned as treatments. For the mycelium and conidia samples of each isolate, three gels from three independent biological replicates were analysed. The DIGE gels were used to analyse the proteins with different levels of abundance, and the 2-DE gels were used to pick these spots for MS identification. The differential in-gel analysis (DIA) module with an estimated spot number of 3000 was employed for spot detection, and the biological variation analysis (BVA) module was used to identify protein spots with different abundances (more than 1.5-fold) that were statistically significant (confidence above 95%, *p* < 0.05) between two races. Only the protein spots with abundance changes at greater than 1.5-fold and that were statistically significant among different samples were manually examined and then selected for identification. The results are shown as the average ± SE (n = 3) in Supporting Information 1.

#### In-gel digestion

The spots of interest were excised manually from the 2-DE gels and washed in 100 μl buffer (50% 100 mM NH_4_HCO_3_, 50% ACN) for 30 min at room temperature with shaking until the gel pieces were transparent. Next, the gels were washed with 150 μL ddH_2_O for 10 min three times. Thereafter, the gel pieces were dried with 100 μl ACN. The air-dried and crystallized spots were digested with trypsin (Roche) at 37 °C overnight^51^. After digestion, the supernatant was collected by centrifugation and used for MS analyses.

#### MS identification and database searches

The peptide mixtures were diluted with the matrix solution, which was generated by dissolving α- cyano-4-hydroxycinnaminic acid (Bruker Daltonics, Billerica, MA, USA) in 50% acetonitrile and 0.1% TFA^33^. The mass spectra were obtained using an Ultraflex MALDI TOF/TOF MS instrument (Bruker Daltonics, Bremen, Germany) and analysed using flexAnalysis software (Version 3.2). The peptide mass fingerprint spectra were internally calibrated with trypsin autolysis peaks; known contaminants were excluded during this process. Next, the measured tryptic peptide messes were transferred through the MS BioTool program (Bruker Daltonics). The data were analysed using a MASCOT search. The proteins were identified using the peptide masses or peptide fragments generated via MALDI-TOF/TOF MS or MALDI-TOF/TOF MS/MS and searched against the fungi taxonomy in the non-redundant NCBI database using the MASCOT search engine (http://www.matrixscience.com). The MASCOT search parameters were as follows: the peptide mass tolerance was 300 ppm; trypsin with 1 missed cleavage; fixed modifications of carbamidomethyl (C); and variable modifications of oxidation (M). In addition, the MS/MS ion search was performed using the above search parameters with an MS/MS tolerance of ±0.5 Da. Proteins with protein score confidence intervals greater than 95% were considered confident identifications. To confirm the matches further, we used a BLAST search at the NCBI. To generate functional information on these proteins, we conducted a BLAST-P search for homologues using the NCBI accession numbers. The identified proteins were further classified into different groups based on their corresponding COG codes, which were obtained using a COG analysis at the NCBI. And the GO pathway analysis was also conducted using the GO annotation search tool in the NCBI. Furthermore, the subcellular locations of the identified proteins were also performed using TargetP. Detailed information on the identified proteins, including functional classification, subcellular locations and GO annotation, is also shown in Supporting Information 1.

#### Quantitative real-time PCR

To further evaluate the identified proteins in proteomic analysis, quantitative real-time PCR (qRT-PCR) was performed to analyse their expression patterns in the R1 and R2 mycelium and conidia. RNA extraction, cDNA synthesis and qPCR were performed as described by McGrath using a Bio-Rad CFX Real-Time PCR System^52^. The *β-tubulin* was used as an internal control to compare the expression levels of 20 typical genes in the mycelium and conidia between R1 and R2. The *β-tubulin* gene primers were designed from FOXG_06228 in *F. oxysporum* f. sp. *lycopersici* and have been previously verified for use in another isolate *F. oxysporum* Fo5176, which was isolated from *Brassica oleracea* plants^53^. The qRT-PCR reactions for each gene were biologically repeated four times. The primers were tested for expected size and gene specificity for the PCR product. The primer sequences are listed in Supporting Information 2: Table S2.

#### *Gas1* deletion in *F. oxysporum* f. sp conglutinans

The *gas1* gene was deleted by overlap PCR and split marker gene strategy. The strategy used a DNA mixture containing overlapping truncations of the selectable marker to integrate homologues in transformation^54^,^55^,^56^,^57^. The 1.4 kb fragment that encoded hygromycin B phosphotransferase as a selectable marker was used to replace the 1656 bp targeted gene. A 1484 bp region upstream and a 1324 bp region downstream from the targeted gene were amplified from Foc genomic DNA using the primers 1-F/(2 + 5)-R and (8 + 3)-F/4-R. The 3’ of upstream fragment and 5’ of downstream fragment were introduced at a marker tail site (Figure S2). The split replacement gene was amplified from the vector PCH-SGFP carrying *hph* gene^58^. The two split marker transformation components were amplified through two round PCR. The steps are shown in Supporting Information 2: Figure S2. The PCR mixtures of the two split marker transformation components were used directly for protoplast-mediated transformation. The right deletion mutants (Foc-∆gas1) were confirmed by PCR (Figure S1 of Supporting Information 2). Moreover, they were also assessed through Southern blotting. The genomic DNA was digested with *Hind*III, separated on a 0.8% agarose gel, and hybridized with a 547 bp probe. The primers related to gene deletion and used to ensure the correct deletion mutants are listed in Table S3 of Supporting Information 2.

## Additional Information

**How to cite this article**: Li, E. *et al*. Comparative Proteomics Analyses of Two Races of *Fusarium oxysporum* f. sp. *conglutinans* that Differ in Pathogenicity. *Sci. Rep*. **5**, 13663; doi: 10.1038/srep13663 (2015).

## Supplementary Material

Supplementary Table S1

Supplementary Information

## Figures and Tables

**Table 1:**
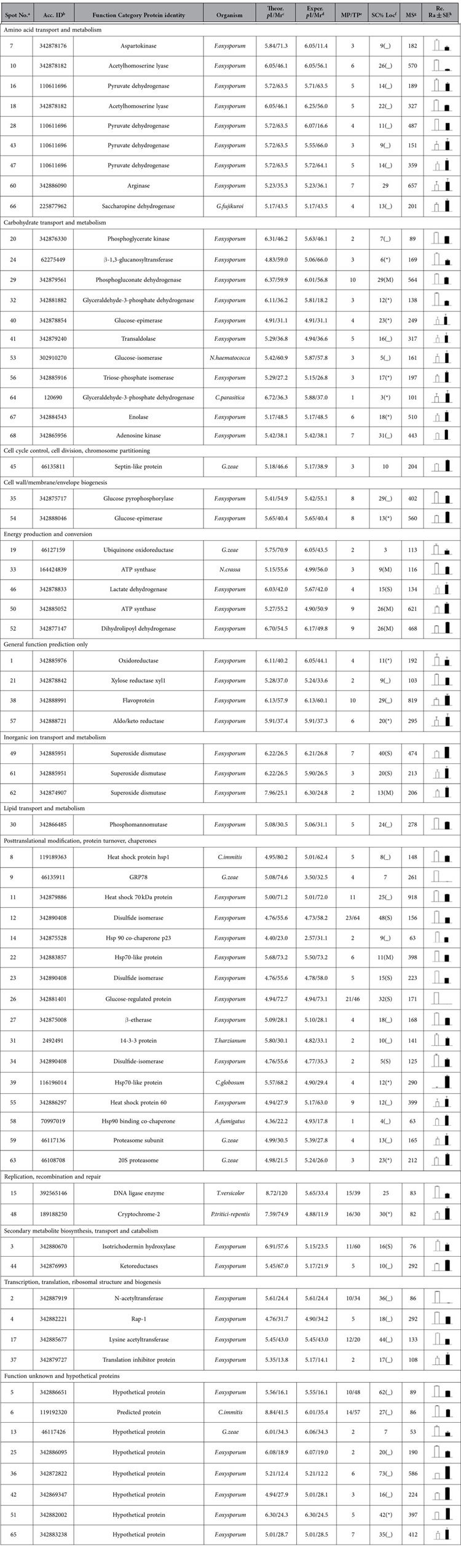
The identified proteins with significantly different abundances between the R1 and R2 mycelium.

^a^The designated spot number as indicated in Table 1 and marked on the DIGE gels in Fig. 2;

^b^the accession numbers for the NCBInr database;

^c^theoretical mass (kDa) and *p*I of proteins identified from the database;

^d^experimental mass and *p*I of the identified proteins. The experimental values were calculated using the Image Master 2D Platinum software and standard molecular mass markers;

^e^matched number of peptides (MP) identified from PFF or PMF data and the total searched peptides (TP);

^f^the amino acid sequence coverage (SC) for the identified proteins and the protein location (Loc) predicted by TargetP;

^g^the Mascot score (MS) from the search against the NCBInr database;

^h^the mean value of the relative protein ratio on the DIGE gels. White represents R1, and black represents R2.

**Table 2:**
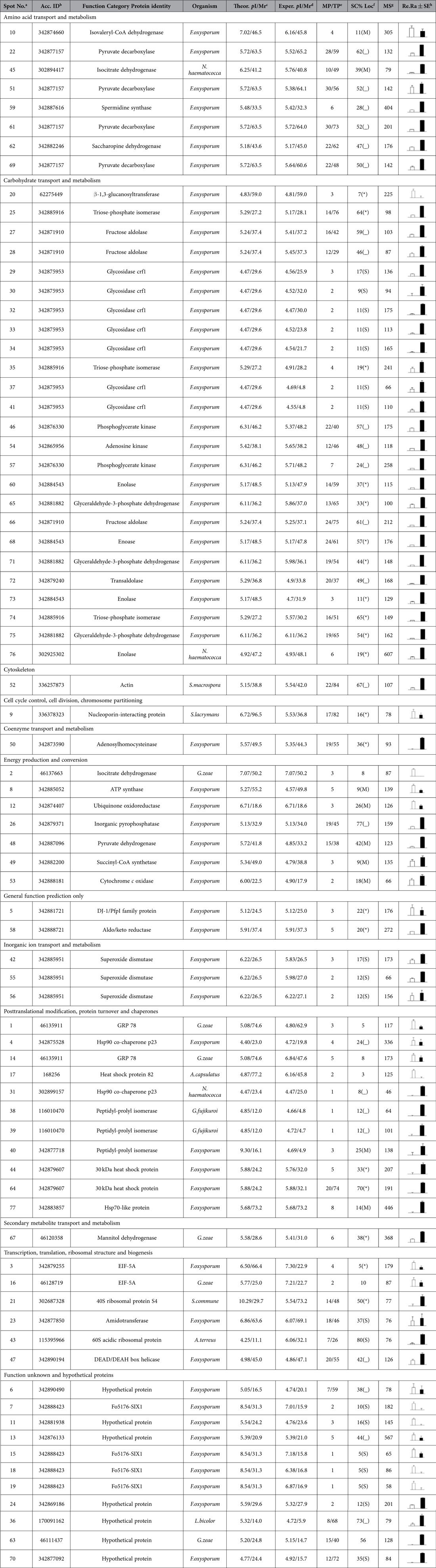
Identification of proteins with significantly different abundances in the R1 and R2 conidia.

^a^The designated spot number as indicated in Table 2 and marked on the DIGE gels in Fig. 3;

^b^the accession numbers for the NCBInr database;

^c^theoretical mass (kDa) and *p*I of identified proteins from the database;

^d^experimental mass and *p*I of the identified proteins. The experimental values were calculated using the Image Master 2D Platinum software and standard molecular mass markers;

^e^matched number of peptides (MP) identified from PFF or PMF data and the total searched peptides (TP);

^f^The amino acid sequence coverage (SC) for the identified proteins and the protein location (Loc) predicted by TargetP;

^g^the Mascot score (MS) from the search against the NCBInr database;

^h^the mean value of the relative protein ratio on the DIGE gels. White represents R1, and black represents R2.
