# Supplementary material for: Comparative Proteomics Analyses of Two Races of Fusarium oxysporum f. sp. conglutinans that Differ in Pathogenicity
Source: Sci Rep. 2015 Sep 3;5:13663. doi: 10.1038/srep13663 (PMC4642580; doi:10.1038/srep13663)
Supplement: Supplementary Information [file srep13663-s2.doc]

**Comparative Proteomics Analyses** **of Two Races of**

***Fusarium oxysporum* f. sp. *conglutinans* that Differ in Pathogenicity**

Erfeng Li^1#^, Jian Ling^1#^, Gang Wang^1#^, Jiling Xiao^1^, Yuhong Yang^1^, Zhenchuan Mao^1^,

Xuchu Wang^2*^, Bingyan Xie^1*^

1. The Institute of Vegetables and Flowers, Chinese Academy of Agricultural Sciences, Beijing 100081, China

2. The Institute of Tropical Biosciences and Biotechnology, Chinese Academy of Tropical Agricultural Sciences, Haikou Hainan 571101, China

^#^ The authors contributed equally to this work.

^*^Corresponding authors:

Dr. Bingyan Xie

Tel.: +86 010 82109545; Fax: +86 010 82109545

E-mail address: lrfcaas@gmail.com

Dr. Xuchu Wang

Tel.: +86 898 66987460; Fax: +86 898 66987460

E-mail address: xchwanghainan@163.com

**
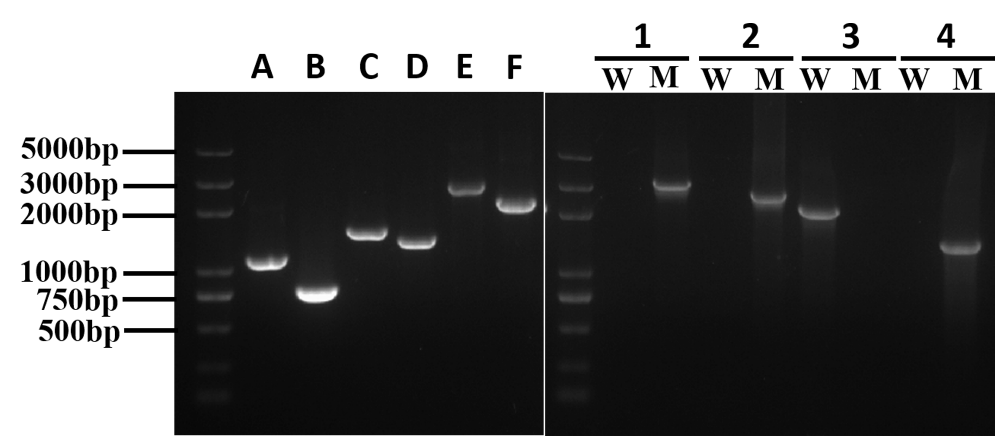
**

**Figure S1** The construction and verification of deletion mutant Foc-∆gas1. A and B. The split replacements of *hph* gene; C and D. The upstream and downstream homologous recombination fragments of *gas1* gene; E and F. The fusion fragments between A and C, B and D were directly used for protoplast-mediated transformation; 1, 2, 3, 4. Four pairs of primers used to verify the mutants Foc-∆gas1 for upstream, downstream, target gene and *hph* gene; W. Wild type isolate; M. Mutant.

**Table S1 Design of 2-D DIGE experiments**

| **Gel number** | **Cy2**  **(internal standard)** | **Cy3**  **(R1)** | **Cy5**  **(R2)** |
| --- | --- | --- | --- |
| **1** | mixtures of the four samples | mycelium | mycelium |
| **2** | mixtures of the four samples | conidia | conidia |

**Table S2 The primer pairs of 20 typical proteins for qRT-PCR analysis**

| **No.** ***^a^*** | **Spot No.*^b^*** | **Race*^c^*** | **Accession No.*^d^*** | **Protein name** | **Sequences*****^e^*** |
| --- | --- | --- | --- | --- | --- |
| **Conidia** | | | | | |
| **1** | 29,30,32,33,34,37,41 | R2 | [342875953](http://www.matrixscience.com/cgi/protein_view.pl?file=..%2Fdata%2F20121112%2FFtGrOaYOT.dat&hit=gi|342875953&db_idx=1&px=1&ave_thresh=46&_ignoreionsscorebelow=0&report=20&_sigthreshold=0.05&_msresflags=1025&_msresflags2=2&percolate=-1&percolate_rt=0&_minpeplen=7&sessionID=guest_guestsession) | Glycosidase | 5'-GACACCCTCCGCCATCAACT-3'  5'-GGAGCAGCATCGGGAGAACC-3' |
| **2** | 27,28,66 | R2 | 342871910 | Fructose-aldolase | 5'-TACTTCGCTGGCAAGGGTGT-3'  5'-AGCTTCTTGGCGCAGTGGTC-3' |
| **3** | 65,66,75 | R2 | 342881882 | Glyceraldehyde 3-  phosphate dehydrogenase | 5'-AACGGTTTCGGTCGTATCGG-3'  5'-TGAAGATGCCGTGGGAAGAG-3' |
| **4** | 46,57 | R2 | [342876330](http://www.matrixscience.com/cgi/protein_view.pl?file=..%2Fdata%2F20121112%2FFtGrOfcee.dat&hit=gi|342876330&db_idx=1&px=1&ave_thresh=47&_ignoreionsscorebelow=0&report=20&_sigthreshold=0.05&_msresflags=1025&_msresflags2=2&percolate=-1&percolate_rt=0&_minpeplen=7&sessionID=guest_guestsession) | Phosphoglycerate kinase | 5'-ACCGTGCTCACTCCTCCATG-3'  5'-GAAAGCCATACCACCGCAGA-3' |
| **5** | 25,35,74 | R2 | 342885916 | Triose-phosphate isomerase | 5'-CTCCTGCCATCTACCTCCCT-3'  5'-CCAGTTGATGTCGCTGTCCTT-3' |
| **6** | 58 | R2 | 342888721 | Aldo/keto reductase | 5'-GTTACCGTCACCTCGATCTTGC-3'  5'-CTCAGGGCGGTGCTTGTTGT-3' |
| **7** | 60,68,73,  76 | R2 | 342884543 | Enolase | 5'-GTCTTCGCCCGCTCCGTCTA-3'  5'-GGCCTTGGTAACACCCTTGC-3' |
| **8** | 42,55,56 | R2 | 342885951 | Superoxide dismutase | 5'-TACGCCTATGATGCCCTTGA-3'  5'-GTGCCCGCCGCCATTGAACT-3' |
| **9** | 72 | R2 | 342879240 | Transaldolase | 5'-TCTAAGGAGGAGGACCCTGGTG-3'  5'-AACGGGCTCATTCGAGTTCA-3' |
| **10** | 30 | R2 | [342875528](http://www.matrixscience.com/cgi/protein_view.pl?file=..%2Fdata%2F20121112%2FFtGrOrEaS.dat&hit=gi|342875528&db_idx=1&px=1&ave_thresh=47&_ignoreionsscorebelow=0&report=20&_sigthreshold=0.05&_msresflags=1025&_msresflags2=2&percolate=-1&percolate_rt=0&_minpeplen=7&sessionID=guest_guestsession) | Hsp90 co-chaperone | 5'-TGCCAAGAATGTTGAGATAAAGC-3'  5'-TACCGCCAAAGTCACCACCC-3' |
| **11** | 1,14 | R1 | 46135911 | GRP 78 | 5'-GGTAACCGAATCACTCCCTCT-3'  5'-AACTGCTTCTTAGCGCCATC-3' |
| **12** | 7,15,18,  19 | R1 | 342888423 | Fo5176-SIX1 | 5'-TCAAGAGGCTGCGGTTGG-3'  5'-GACGCTCAGGGCGACATA-3' |
| **Mycelium** | | | | | |
| **13** | 56 | R2 | 342885916 | Triose-phosphate isomerase | 5'-CTCCTGCCATCTACCTCCCT-3'  5'-CCAGTTGATGTCGCTGTCCTT-3' |
| **14** | 57 | R2 | 342888721 | Aldo/keto reductase | 5'-GTTACCGTCACCTCGATCTTGC-3'  5'-CTCAGGGCGGTGCTTGTTGT-3' |
| **15** | 67 | R2 | 342884543 | Enolase | 5'-GTCTTCGCCCGCTCCGTCTA-3'  5'-GGCCTTGGTAACACCCTTGC-3' |
| **16** | 49,61 | R2 | 342885951 | Superoxide dismutase | 5'-TACGCCTATGATGCCCTTGA-3'  5'-GTGCCCGCCGCCATTGAACT-3' |
| **17** | 41 | R2 | 342879240 | Transaldolase | 5'-TCTAAGGAGGAGGACCCTGGTG-3'  5'-AACGGGCTCATTCGAGTTCA-3' |
| **18** | 37 | R2 | 342879727 | Translation inhibitor protein | 5'-CGGCCCTTGGCCCTTACTCT-3'  5'-GCCTCAACAACAGCCTGGAC-3' |
| **19** | 9 | R1 | 46135911 | GRP 78 | 5'-GGTAACCGAATCACTCCCTCT-3'  5'-AACTGCTTCTTAGCGCCATC-3' |
| **20** | 12,23,34 | R1 | 342890408 | Disulfide isomerase | 5'-CCTGAACGCCGACGATAAGA-3'  5'-GGTGGCAGAGGTGGTAATGAA-3' |
| **Reference gene** | | | | | |
| ***Foc***  ***Tub*** | - | R1/R2 | FOXG_06228 | Tubulin | 5'-TGTTCGACCCCAAGAACAT-3'  5'-GGTCCTCGACCTCCTTCATA-3' |

a. Designated number as indicated in Figure 6 for qRT-PCR analysis; b. The corresponding spot number marked on the DIGE gels in Figure 2 and Figure 3; c. Races where the highly abundant proteins selected from for qRT-PCR analysis; d.The accession number in NCBInr database; e. The primer sequences for these proteins selected for qRT-PCR analysis.

**
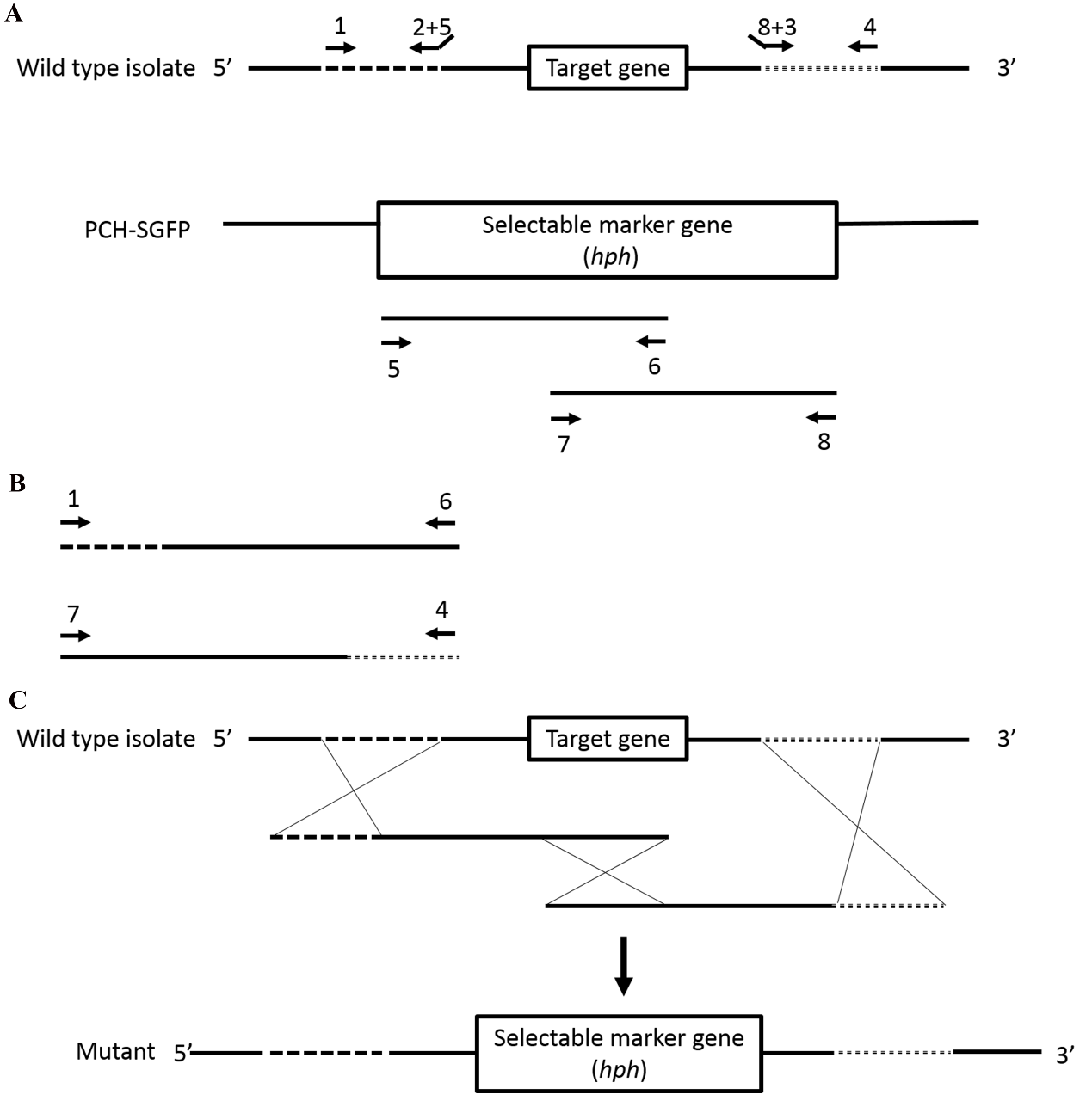
**

**Figure S2** Overlap PCR and split marker gene disruption strategy. A. Amplification of 5’- and 3’- flanking regions of the target gene and split replacement of selectable marker gene; B. Fusion the flanking regions and split marker sequences; C. Homologous recombination between the flank regions of the target gene and their genome counterparts and between the overlapping regions of the selectable marker gene.

**Table S3** Primers for deletion construction using overlap PCR and split marker gene strategy

| **Gene** | **Fragment ^a^** | | **Primers** | **Sequences** |
| --- | --- | --- | --- | --- |
| ***Foc-***  ***gas1*** | A | 5-F  6-R | | 5’-CTTGGCTGGAGCTAGTGGAGGT-3’  5’-GGATGCCTCCGCTCGAAGTA-3’ |
|  | B | 7-F  8-R | | 5’-CGTTGCAAGACCTGCCTGAA-3’  5’-CCCGGTCGGCATCTACTCTATTC-3’ |
|  | C | 1-F  (2+5)-R | | 5’-AGGCTGAGATTTCATAGGCG-3’  5’-ACCTCCACTAGCTCCAGCCAAGCCGGTGCATGGTTTGTTAGT-3’ |
|  | D | (8+3)-F  4-R | | 5’-GAATAGAGTAGATGCCGACCGGGCATAGGGTTTGTAGCGAGGAT-3’  5’-AGTCGGAAGATGTTTGAGGG-3’ |
|  | 1 | Upcheck-F  Upcheck-R | | 5’-TCACCCAAGCACTTCACAGA-3’  5’-GACAGACGTCGCGGTGAGTT-3’ |
|  | 2 | Dcheck-F  Dcheck-R | | 5’-TCTGGACCGATGGCTGTGTAG-3’  5’-TCTGGACCGATGGCTGTGTAG-3’ |
|  | 3 | Gene-F  Gene-R | | 5’-TCATCAAACCTTCGCTAACC-3’  5’-TCACCCAAGCACTTCACAGA-3’ |
|  | 4 | Hph-F  Hph-R | | 5’-CTTGGCTGGAGCTAGTGGAGGT-3’  5’-CCCGGTCGGCATCTACTCTATTC-3’ |
|  |  | Probe-F  Probe-R | | 5’-TTTTCACAACCCGTAACAAG-3’  5’-CTAATGCTTTCAGTGCCTAAT-3’ |

a. The corresponding amplified fragments in Figure S1.

**Supplemental spectra and MALDI TOF/TOF MS/MS identification information for** **Supporting Information 1**

**Annotated spectra for Supporting Information 1:**

**145 proteins identified by MALDI TOF/TOF MS and MALDI TOF/TOF MS/MS**

Spot numbers of the 145 proteins corresponded to the proteins that listed in Supporting Information 1.

**MALDI TOF:**

matrix assisted laser desorption/ionization time of flight

**MS:** mass spectrometry

**PMF:** peptide mass fingerprinting

**PFF:** peptide fragment fingerprinting

**Proteins identified in mycelium of R1**

Spot No.:**1**

NCBI accession No.: **gi|342885976**

Species: ***Fusarium oxysporum* Fo5176**

Protein name**: Oxidoreductase**

**PFF** score**: [192]**

Matched peptides No**.: [4]** Sequence coverage **%: [11]** Matched sequences: **K.LLIVYQNR.R**

**R.RWDADFVTLK.K**

**R.IIQFDNHFDR.Y**

**R.ISVLSAELEQPR.F**

Calculated Mr: **40186** Calculated *p*I: **6.11**

Probability Based Mowse Score**:**

**
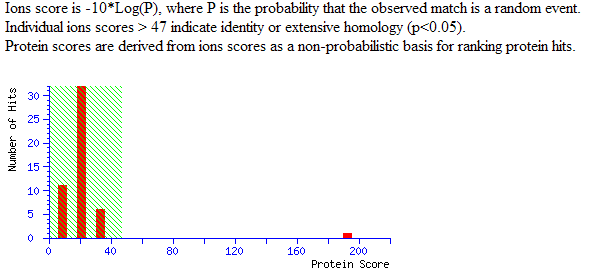
**

Matched peptide sequences: shown in **Bold Red:**

**
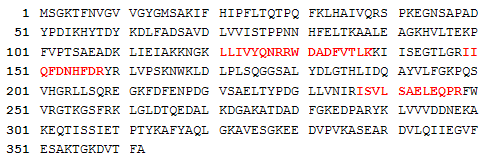
**

Spot No.: **2**

NCBI accession No.: **gi|342887919**

Species: ***Fusarium oxysporum* Fo5176**

Protein name: **Putative N-acetyltransferase**

PMF Mascot score: **86** Sequence coverage %: **36** Matched peptides No.: **10** Total peptides No.: **34**

Calculated Mr: **24433** Calculated *p*I: **5.61**

Probability Based Mowse Score:


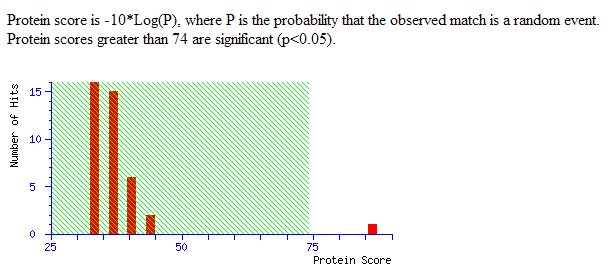


Matched peptide sequences: shown in Bold Red:


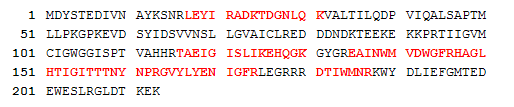


Spot No.: **3**

NCBI accession No.: **gi|342880670**

Species: ***Fusarium oxysporum* Fo5176**

Protein name: **Isotrichodermin C-15 hydroxylase**

PMF Mascot score: **76** Sequence coverage %: **16** Matched peptides No.: **11** Total peptides No.: **60**

Calculated Mr: **57610** Calculated *p*I: **6.91**

Probability Based Mowse Score:


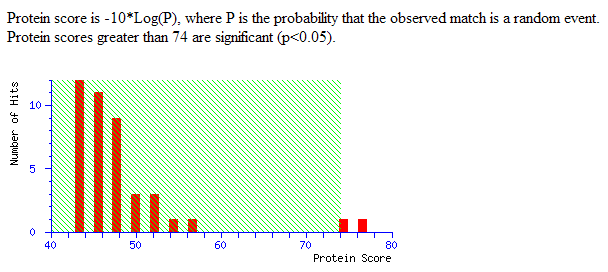


Matched peptide sequences: shown in Bold Red:


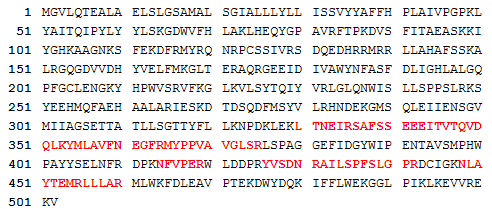


Spot No.:**4**

NCBI accession No.: **gi|342882221**

Species: ***Fusarium oxysporum* Fo5176**

Protein name: **Ribosome-associated protein S2 (Rap-1)**

PFF score: **[292]**

Matched peptides No.: **[5]**  Sequence coverage %: **[18]** Matched sequences: **K.NLQVHMENYLWK.T**

**K.NLQVHMENYLWK.T + Oxidation (M)**

**R.IIAAVDNPADICVISAR.P**

**K.FAAHTGATAIAGR.F**

**R.FTPGSFTNYITR.S**

Calculated Mr: **31737**  Calculated *p*I: **4.76**

Probability Based Mowse Score:


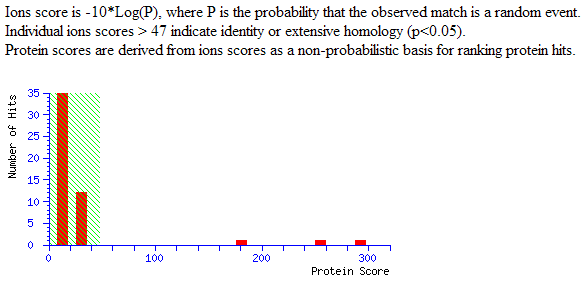


Matched peptide sequences: shown in Bold Red:


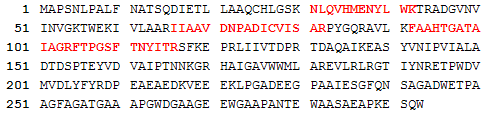


Spot No.: **5**

NCBI accession No.: **gi|342886651**

Species: ***Fusarium oxysporum* Fo5176**

Protein name: **Hypothetical protein FOXB_03062**

PMF Mascot score: **89** Sequence coverage %: **62** Matched peptides No.: **10** Total peptides No.: **48**

Calculated Mr: **16137** Calculated *p*I: **5.56**

Probability Based Mowse Score:


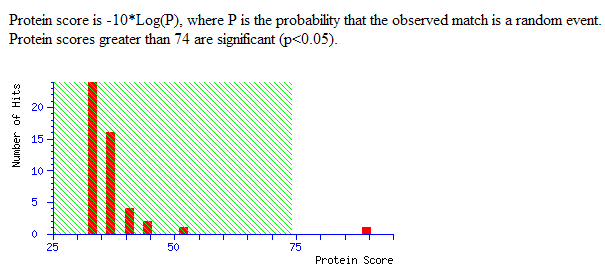


Matched peptide sequences: shown in Bold Red:


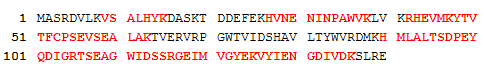


Spot No.: **6**

NCBI accession No.: **gi|119192320**

Species: ***Coccidioides immitis* RS**

Protein name: **Predicted protein**

PMF Mascot score: **86** Sequence coverage %: **27** Matched peptides No.: **14** Total peptides No.: **57**

Calculated Mr: **41545** Calculated *p*I: **8.84**

Probability Based Mowse Score:


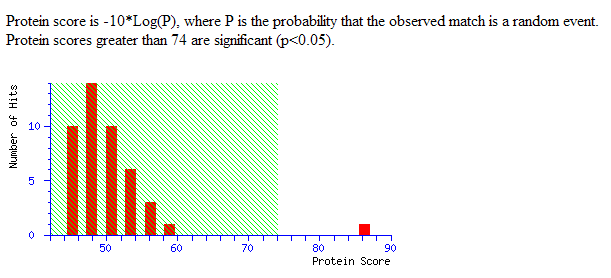


Matched peptide sequences: shown in Bold Red:


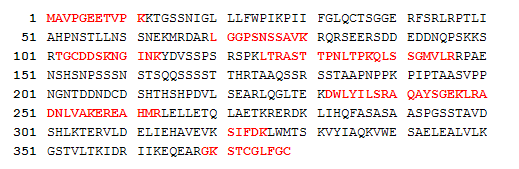


Spot No.: **7**

NCBI accession No.: **gi|342878176**

Species: ***Fusarium oxysporum* Fo5176**

Protein name: **Aspartokinases**

PFF score: **[182]**

Matched peptides No.: **[3]**  Sequence coverage %: **[9]** Matched sequences: **K.EYLVLIPDLPDVLAK.R**

**R.QVLLKPHNQDAAPLVK.A**

**R.VPFFGSTLAHHSAEGQQVAENGTVMIIK.A**Calculated Mr: **71299** Calculated *p*I: **5.84**

Probability Based Mowse Score:


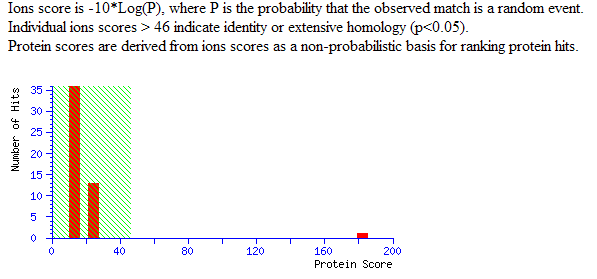


Matched peptide sequences: shown in Bold Red:


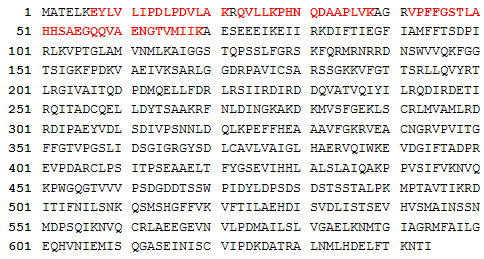


Spot No.: **8**

NCBI accession **No.:** **gi|119189363**

Species: ***Coccidioides immitis* RS**

Protein name: **Heat shock protein hsp1**

PFF score: **[148]**

Matched peptides No.: **[5]**  Sequence coverage %: **[8]** Matched sequences: **K.HFSVEGQLEFR.A**

**K.RAPFDLFETK.K**

**K.GVVDSEDLPLNLSR.E**

**K.LGIHEDSQNR.Q**

**R.TGQFGWSANMER.I**

Calculated Mr: **80162**  Calculated *p*I: **4.95**

Probability Based Mowse Score:


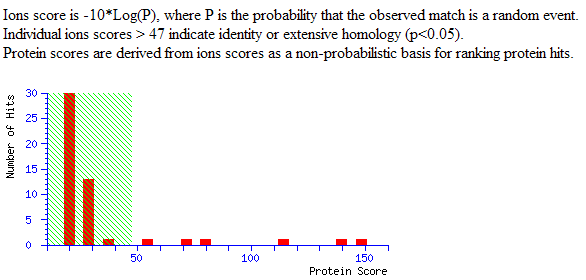


Matched peptide sequences: shown in Bold Red:


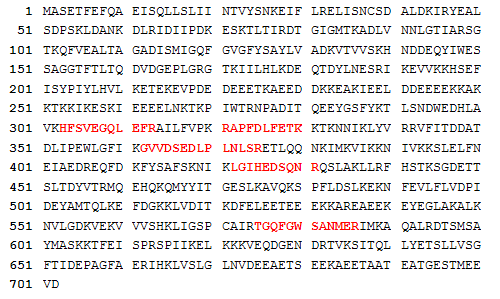


Spot No.: **9**

NCBI accession No.: **gi|46135911**

Species: ***Gibberella zeae* PH-1**

Protein name: **GR78_Neucr 78 KDa glucose-regulated protein homolog precursor (GRP 78)**

PFF score: **[261]**

Matched peptides No.: **[4]**  Sequence coverage %: **[7]** Matched sequences: **R.KSQIFSTAADNQPVVLIQVFEGER.S**

**K.SQIFSTAADNQPVVLIQVFEGER.S**

**K.FELTGIPPAPR.G**

**R.GVPQIEVSFELDANGILK.V**

Calculated Mr: **74638**  Calculated *p*I: **5.08**

Probability Based Mowse Score:


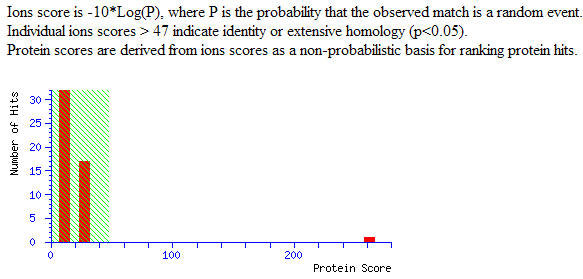


Matched peptide sequences: shown in Bold Red:


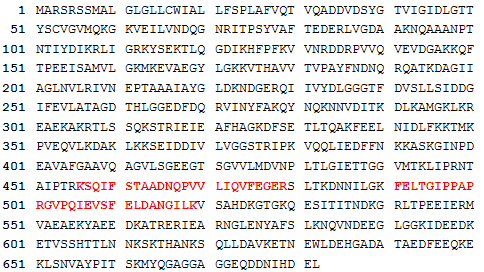


Spot No.: **10**

NCBI accession No.: **gi|342878182**

Species: ***Fusarium oxysporum* Fo5176**

Protein name: **O-acetylhomoserine (thiol)-lyase**

PFF score: **[570]**

Matched peptides No.: **[6]**  Sequence coverage %: **[26]** Matched sequences: **R.STAVPIYATSSYTFNDSAHGAR.L**

**K.AGDNIVASSHLYGGTYNQLNVLLPR.F**

**K.LEDYAAAIDDQTR.A**

**K.WIGGHGTTIGGVIVDSGR.F**

**K.YWEAFGPATFITR.I**

**K.SLAIHPWSTTHEQLSEDER.L**

Calculated Mr: **46101** Calculated *p*I: **6.05**

Probability Based Mowse Score:


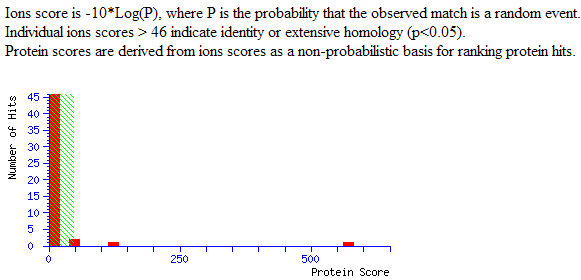


Matched peptide sequences: shown in Bold Red:


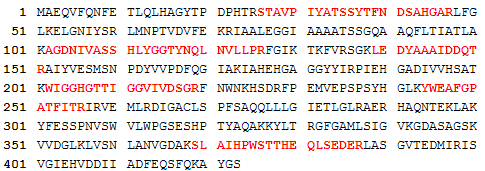


Spot No.: **11**

NCBI accession No.: **gi|342879886**

Species: ***Fusarium oxysporum* Fo5176**

Protein name: **Heat shock 70 kDa protein**

PFF score: **[918]**

Matched peptides No.: **[11]**  Sequence coverage %: **[25]** Matched sequences: **M.APAVGIDLGTTYSCVGIFR.E**

**R.ETAESYLGETVNNAVVTVPAYFNDSQR.Q**

**K.STAGDTHLGGEDFDNR.L**

**R.LVNHFVNEFK.R**

**R.FEELCQDLFR.S**

**K.SLVHEIVLVGGSTR.I**

**K.KSEVFSTFSDNQPGVLIQVYEGER.Q**

**K.SEVFSTFSDNQPGVLIQVYEGER.Q**

**K.FELTGIPPAPR.G**

**R.GVPQIEVTFDLDANGIMNVSAVEK.G**

**K.VVQWLDDNQQATR.E**

Calculated Mr: **71192** Calculated *p*I: **5.00**

Probability Based Mowse Score:


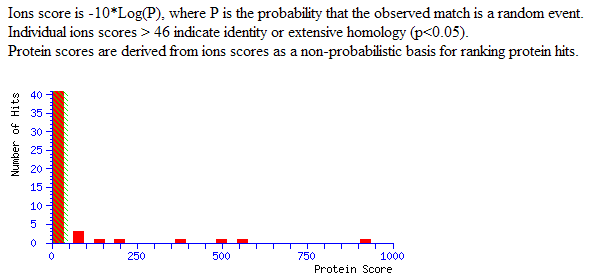


Matched peptide sequences: shown in Bold Red:


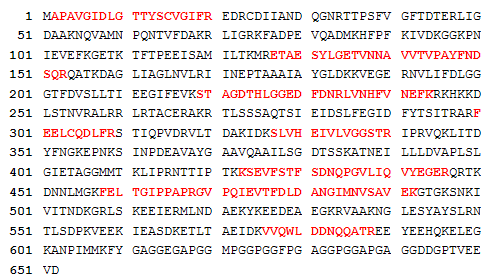


Spot No.: **12**

NCBI accession No.: **gi|342890408**

Species: ***Fusarium oxysporum* Fo5176**

Protein name: **Protein disulfide-isomerase**

PMF Mascot score: **156** Sequence coverage %: **48** Matched peptides No.: **23** Total peptides No.: **64**

Calculated Mr: **55623** Calculated *p*I: **4.76**

Probability Based Mowse Score:


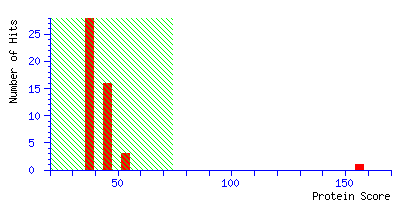


Matched peptide sequences: shown in Bold Red:


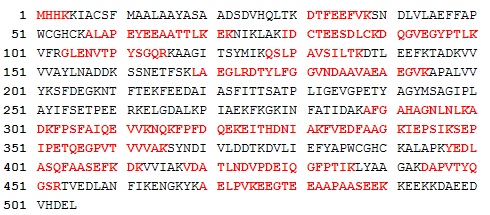


Spot No.: **13**

NCBI accession No.: **gi|46117426**

Species: ***Gibberella zeae* PH-1**

Protein name: **Hypothetical protein FG04555.1**

PFF score: **[53]**

Matched peptides No.: **[2]** Sequence coverage %: **[7]** Matched sequences: **K.QLHASHVSTYDR.L**

**R.HGLDVPLGENR.E**

Calculated Mr: **34281** Calculated *p*I: **6.01**

Probability Based Mowse Score:


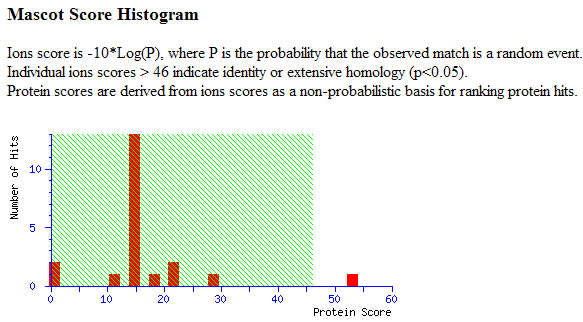


Matched peptide sequences: shown in Bold Red:


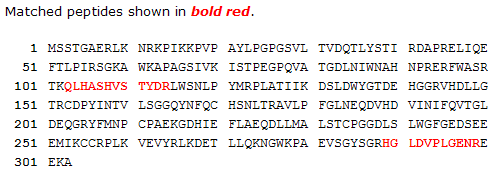


Spot No.:**14**

NCBI accession No.: **gi|342875528**

Species: ***Fusarium oxysporum* Fo5176**

Protein name: **Hsp90 associated co-chaperone**

PFF score: **[63]**

Matched peptides No.: **[2]**  Sequence coverage %: **[9]** Matched sequences: **K.LTFTGTSSTLK.K**

**K.ELKEEYWPR.L**

Calculated Mr: **23030** Calculated *p*I: **4.40**

Probability Based Mowse Score:


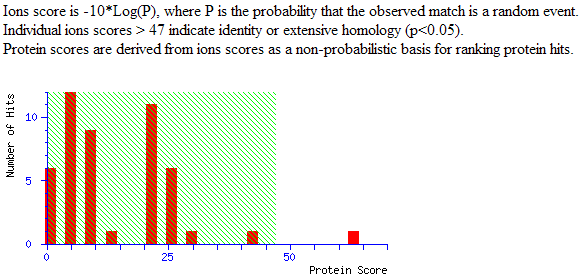


Matched peptide sequences: shown in Bold Red:


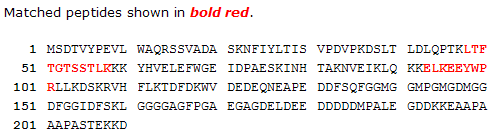


Spot No.:**15**

NCBI accession No.: **gi|392565146**

Species: ***Trametes versicolor* FP-101664 SS1**

Protein name: **DNA ligase/mRNA capping enzyme**

PMF Mascot score: **83** Sequence coverage %: **25** Matched peptides No.: **15** Total peptides No.: **39**

Calculated Mr: **120378** Calculated *p*I: **8.72**

Probability Based Mowse Score:


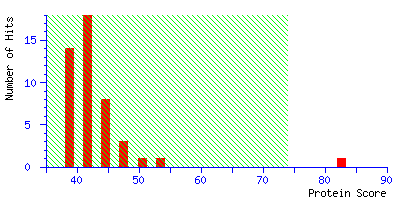


Matched peptide sequences: shown in Bold Red:


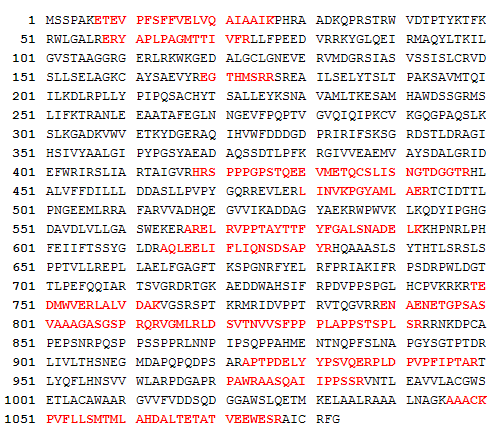


Spot No.: **16**

NCBI accession No.: **gi|110611696**

Species: ***Fusarium oxysporum* f. sp. *lycopersici***

Protein name: **Putative pyruvate decarboxylase**

PFF score: **[189]**

Matched peptides No.: **[5]**  Sequence coverage %: **[14]** Matched sequences: **K.WVGSVNELNAAYAADGYAR.V**

**K.GAVNEDHPNYGGVFAGDGSHPAR.A**

**K.SDFNTTGFSYR.T**

**R.TSQINSVDFHSTHCK.V**

**K.NTDDSEIITQAWLWPR.V**

Calculated Mr: **63493** Calculated *p*I: **5.72**

Probability Based Mowse Score:


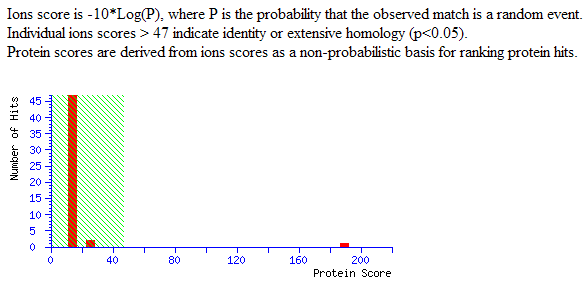


Matched peptide sequences: shown in Bold Red:


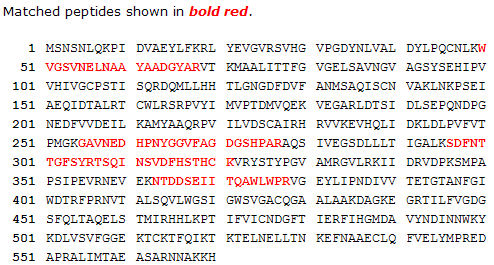


Spot No.: **17**

NCBI accession No.: **gi|342885677**

Species: ***Fusarium oxysporum* Fo5176**

Protein name: **Lysine acetyltransferase**

PMF Mascot score: **133** Sequence coverage %: **44** Matched peptides No.: **12** Total peptides No.: **40**

Calculated Mr: **43008** Calculated *p*I: **5.45**

Probability Based Mowse Score:


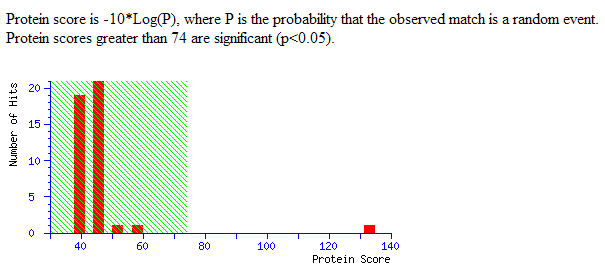


Matched peptide sequences: shown in Bold Red:


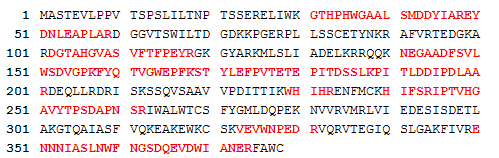


Spot No.: **18**

NCBI accession No.: **gi|342878182**

Species: ***Fusarium oxysporum* Fo5176**

Protein name: **O-acetylhomoserine (thiol)-lyase**

PFF score: **[327]**

Matched peptides No.: **[5]**  Sequence coverage %: **[22]** Matched sequences: **R.STAVPIYATSSYTFNDSAHGAR.L**

**K.AGDNIVASSHLYGGTYNQLNVLLPR.F**

**K.WIGGHGTTIGGVIVDSGR.F**

**K.YWEAFGPATFITR.I**

**K.SLAIHPWSTTHEQLSEDER.L**

Calculated Mr: **46101** Calculated *p*I: **6.05**

Probability Based Mowse Score:


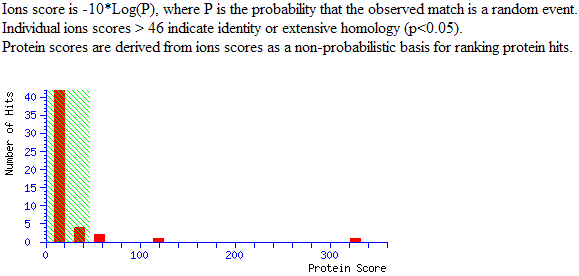


Matched peptide sequences: shown in Bold Red:


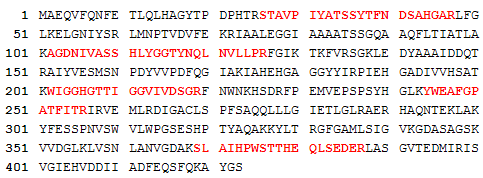


Spot No.: **19**

NCBI accession No.: **gi|46127159**

Species: ***Gibberella zeae* PH-1**

Protein name: **NADH-ubiquinone oxidoreductase 39 kda**

PFF score: **[113]**

Matched peptides No.: **[2]**  Sequence coverage %: **[3]** Matched sequences: **R.VVFIEHDLR.N**

**K.NFSLEDVHVEGTER.I**

Calculated Mr: **70852** Calculated *p*I: **5.75**

Probability Based Mowse Score:


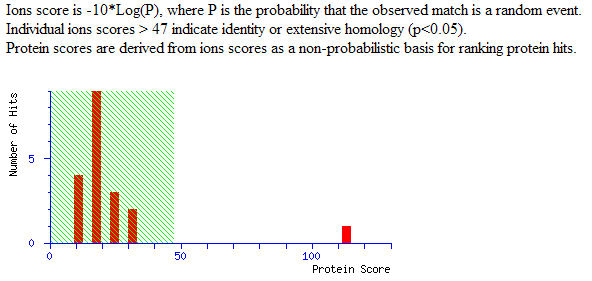


Matched peptide sequences: shown in Bold Red:


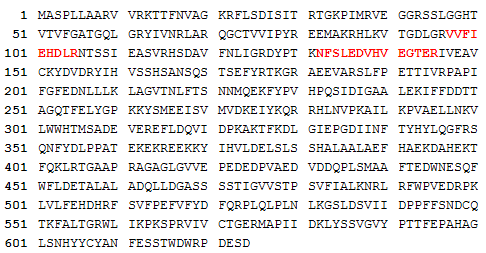


Spot No.: **20**

NCBI accession No.: **gi|342876330**

Species: ***Fusarium oxysporum* Fo5176**

Protein name: **Phosphoglycerate kinase**

PFF score: **[89]**

Matched peptides No.: **[2]**  Sequence coverage %: **[7]** Matched sequences: **K.AEDGAVILLENLR.F**

**K.GLTALGDVYINDAFGTAHR.A**

Calculated Mr: **46156** Calculated *p*I: **6.31**

Probability Based Mowse Score:


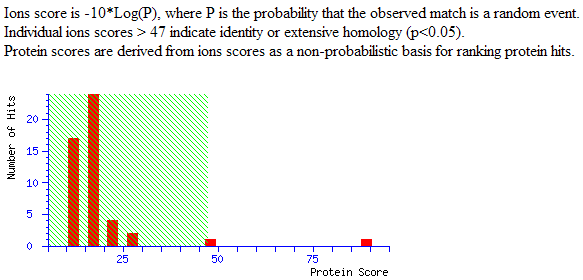


Matched peptide sequences: shown in Bold Red:


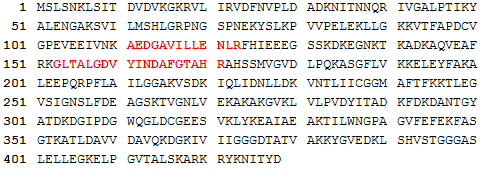


Spot No.: **21**

NCBI accession No.: **gi|342878842**

Species: ***Fusarium oxysporum* Fo5176**

Protein name: **NAD(P)H-dependent D-xylose reductase xyl1**

PFF score: **[103]**

Matched peptides No.: **[2]**  Sequence coverage %: **[9]** Matched sequences: **K.LWQTYHDKENVEPITR.R**

**R.YPPGWHYDDAGTEIR.W**

Calculated Mr: **36958** Calculated *p*I: **5.28**

Probability Based Mowse Score:


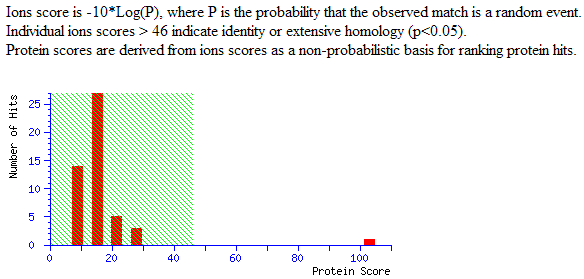


Matched peptide sequences: shown in Bold Red:


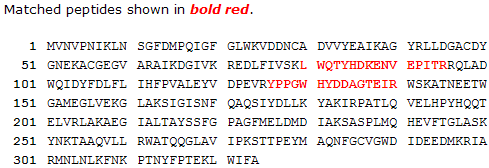


Spot No.: **22**

NCBI accession No.: **gi|342883857**

Species: ***Fusarium oxysporum* Fo5176**

Protein name: **Hsp70-like protein**

PFF score: **[398]**

Matched peptides No.: **[6]** Sequence coverage %: **[11]** Matched sequences: **K.IVQHTNGDAWVAAR.G**

**K.NAVVTVPAYFNDSQR.Q**

**K.EIQEVILVGGMTR.M**

**K.EIQEVILVGGMTR.M + Oxidation (M)**

**K.LLGNFQLVGIPPAHR.G**

**R.GVPQVEVTFDIDADSIVHVHAK.D**

Calculated Mr: **73180**  Calculated *p*I: **5.68**

Probability Based Mowse Score:


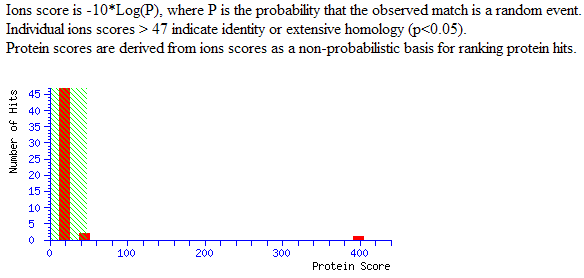


Matched peptide sequences: shown in Bold Red:


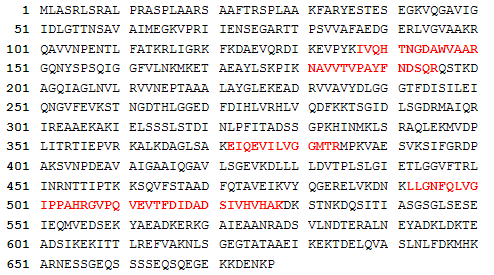


Spot No.:**23**

NCBI accession No.: **gi|342890408**

Species: ***Fusarium oxysporum* Fo5176**

Protein name: **Protein disulfide-isomerase**

PFF score: **[223]**

Matched peptides No.: **[5]**  Sequence coverage %: **[15]** Matched sequences: **R.DTYLFGGVNDAAVAEAEGVK.A**

**K.AFGAHAGNLNLK.A**

**K.ADKFPSFAIQEVVK.N**

**K.YEDLASQFAASEFK.D**

**K.VDATLNDVPDEIQGFPTIK.L**

Calculated Mr: **55623** Calculated *p*I: **4.76**

Probability Based Mowse Score:


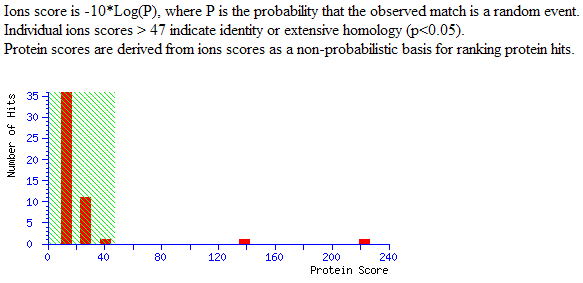


Matched peptide sequences: shown in Bold Red:


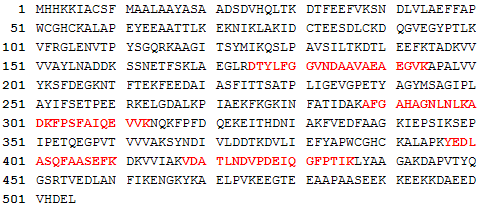


Spot No.: **24**

NCBI accession No.: **gi|62275449**

Species: ***Fusarium oxysporum***

Protein name: **Beta-1, 3-glucanosyltransferase**

PFF score: **[169]**

Matched peptides No.: **[3]** Sequence coverage %: **[6]** Matched sequences: **K.WDVELYER.Y**

**R.EQIADYFNCGDDDSR.I**

**R.IFDETTALYEEK.V**

Calculated Mr: **59047** Calculated *p*I: **4.83**

Probability Based Mowse Score:


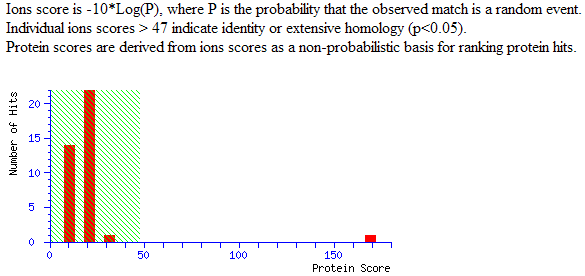


Matched peptide sequences: shown in Bold Red:


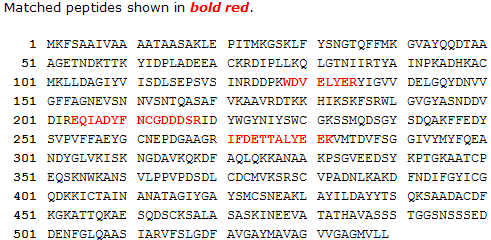


Spot No.:**25**

NCBI accession No.: **gi|342886095**

Species: ***Fusarium oxysporum* Fo5176**

Protein name: **Hypothetical protein FOXB_03437**

PFF score: **[190]**

Matched peptides No.: **[2]** Sequence coverage %: **[20]** Matched sequences: **R.DECTTFPQFHAR.I**

**R.DYVQGYGIPNVFFHLTTAYAILR.K**

Calculated Mr: **18908** Calculated *p*I: **6.08**

Probability Based Mowse Score:


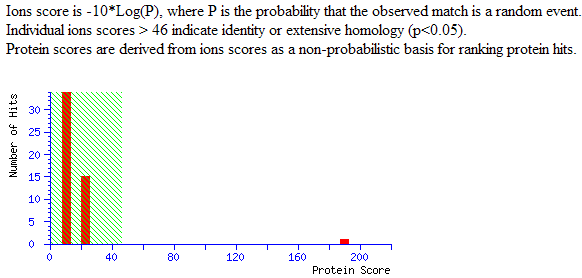


Matched peptide sequences: shown in Bold Red:


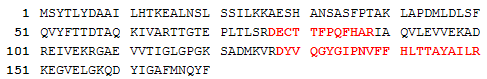


Spot No.: **26**

NCBI accession No.: **gi|342881401**

Species: ***Fusarium oxysporum* Fo5176**

Protein name: **Glucose-regulated protein 78 of hsp70 family**

PMF Mascot score: **171** Sequence coverage %: **32** Matched peptides No.: **21** Total peptides No.: **46**

Calculated Mr: **72662** Calculated *p*I: **4.94**

Probability Based Mowse Score:


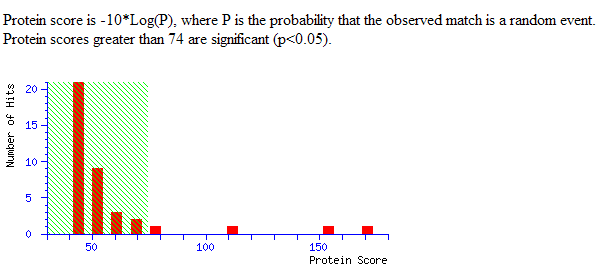


Matched peptide sequences: shown in Bold Red:


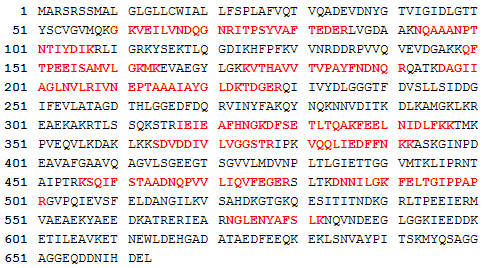


Spot No.:**27**

NCBI accession No.: **gi|342875008**

Species: ***Fusarium oxysporum* Fo5176**

Protein name: **Beta-etherase**

PFF score: **[168]**

Matched peptides No.: **[4]**  Sequence coverage %: **[18]** Matched sequences: **K.NICFSPVVWK.I**

**R.DEGPFLLGEKPSYTDFFIAASLQSSR.T**

**K.PSYTDFFIAASLQSSR.T**

**R.TIDDGIFQR.C**

Calculated Mr: **28118** Calculated *p*I: **5.09**

Probability Based Mowse Score:


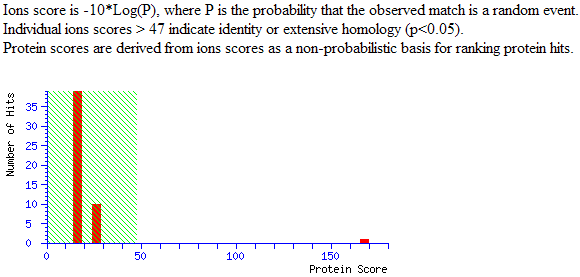


Matched peptide sequences: shown in Bold Red:


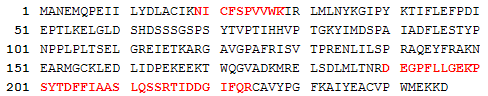


Spot No.: **28**

NCBI accession No.: **gi|110611696**

Species: ***Fusarium oxysporum* f. sp. *lycopersici***

Protein name: **Putative pyruvate decarboxylase**

PFF score: **[487]**

Matched peptides No.: **[4]** Sequence coverage %: **[11]** Matched sequences: **K.PIDVAEYLFK.R**

**R.SVHGVPGDYNLVALDYLPQCNLK.W**

**K.WVGSVNELNAAYAADGYAR.V**

**K.LNKPSEIAEQIDTALR.T**

Calculated Mr: **63493** Calculated *p*I: **5.72**

Probability Based Mowse Score:


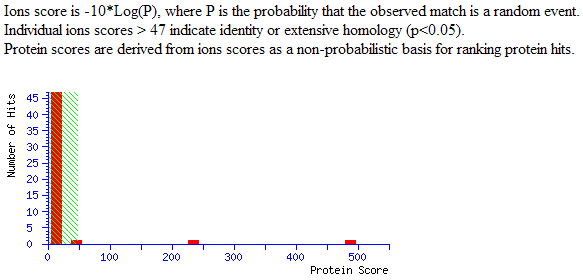


Matched peptide sequences: shown in Bold Red:


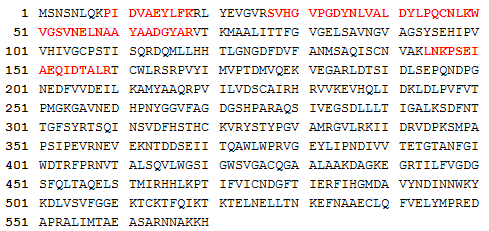


Spot No.:**29**

NCBI accession No.: **gi|342879561**

Species: ***Fusarium oxysporum* Fo5176**

Protein name: **6-phosphogluconate dehydrogenase**

PFF score: **[564]**

Matched peptides No.: **[10]**  Sequence coverage %: **[29]** Matched sequences: **K.ILPLLDAGDIIIDGGNSHFPDSNR.R**

**R.FVGSGVSGGEEGAR.Y**

**K.SDGEACCEWVGDEGAGHYVK.M**

**K.GVLDSFLIEITR.D**

**K.WTAVNALDLGQPVTLIAEAVLAR.C**

**K.LNKPSIALMWR.G + Oxidation (M)**

**R.SQPDLQNLLFDDFFNK.A**

**K.AALLGIPTPAFSTALSWFDGYR.T**

**K.DLPANLLQAQR.D**

**R.DYFGAHTFR.I**

Calculated Mr: **59948** Calculated *p*I:**6.37**

Probability Based Mowse Score:


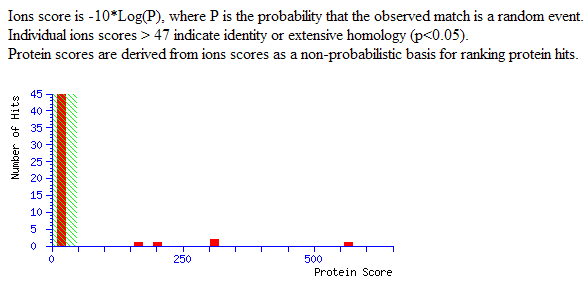


Matched peptide sequences: shown in Bold Red:


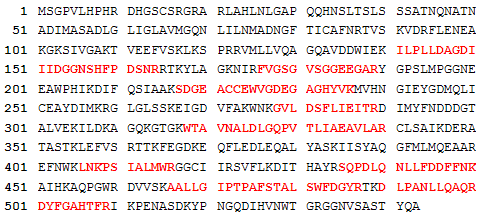


Spot No.: **30**

NCBI accession No.: **gi|342866485**

Species: ***Fusarium oxysporum* Fo5176**

Protein name: **Phosphomannomutase**

PFF score: **[278]**

Matched peptides No.: **[5]**  Sequence coverage %: **[24]** Matched sequences: **K.DTICLFDVDGTLTPAR.L**

**K.CAIGYVGGSDFAK.Q**

**R.NDFEAFDKDAK.V**

**K.TYCLQHLENEAK.K**

**K.TFEGGNDYEIYTDSR.T**

Calculated Mr: **30542** Calculated *p*I: **5.08**

Probability Based Mowse Score:


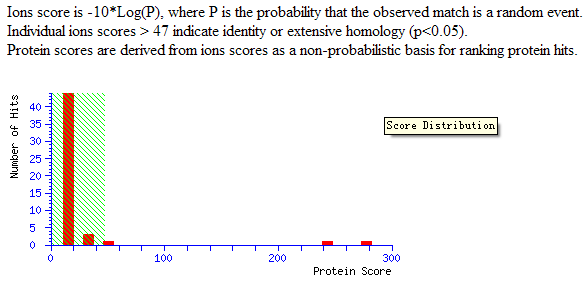


Matched peptide sequences: shown in Bold Red:


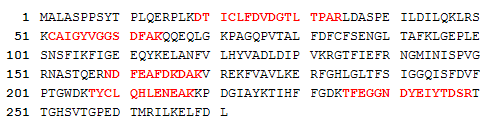


Spot No.: **31**

NCBI accession No.: **gi|2492491**

Species: ***Trichoderma harzianum***

Protein name: **14-3-3 protein homolog**

PFF score: **[141]**

Matched peptides No.: **[2]**  Sequence coverage %: **[10]** Matched sequences: **R.YLAEFAIGDR.R**

**K.AATEVAQTELPPTHPIR.L**

Calculated Mr: **30094**  Calculated *p*I: **5.80**

Probability Based Mowse Score:


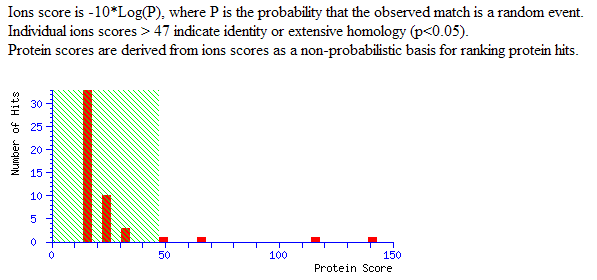


Matched peptide sequences: shown in Bold Red:


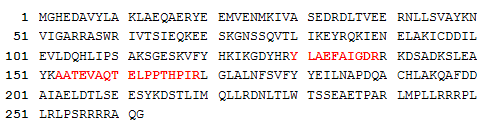


Spot No.: **32**

NCBI accession No.: **gi|342881882**

Species: ***Fusarium oxysporum* Fo5176**

Protein name: **Glyceraldehyde-3-phosphate dehydrogenase**

PFF score: **[138]**

Matched peptides No.: **[3]**  Sequence coverage %: **[12]** Matched sequences: **K.YDSSHGIFK.G**

**K.FYSERDPANIK.W**

**K.WSETGADYVVESTGVFTTTEK.A**

Calculated Mr: **36213** Calculated *p*I: **6.11**

Probability Based Mowse Score:


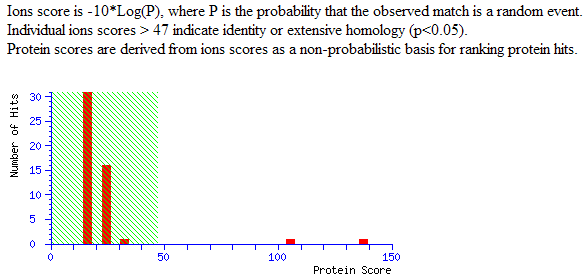


Matched peptide sequences: shown in Bold Red:


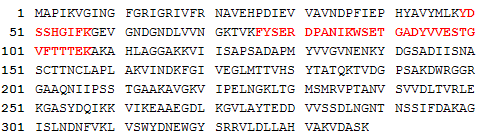


Spot No.:**33**

NCBI accession No.: **gi|164424839**

Species: ***Neurospora crassa* OR74A**

Protein name: **ATP synthase beta chain, mitochondrial precursor**

PFF score: **[116]**

Matched peptides No.: **[3]**  Sequence coverage %: **[9]** Matched sequences: **R.GAKASDTGAPITIPVGPATLGR.I**

**K.AHGGYSVFTGVGER.T**

**K.VALVFGQMNEPPGAR.A**

Calculated Mr: **55556** Calculated *p*I: **5.15**

Probability Based Mowse Score:


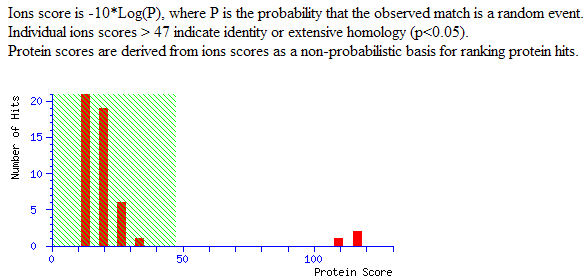


Matched peptide sequences: shown in Bold Red:


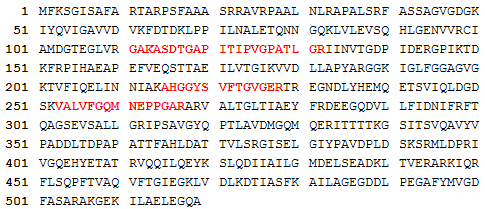


Spot No.: **34**

NCBI accession No.: **gi|342890408**

Species: ***Fusarium oxysporum* Fo5176**

Protein name: **Protein disulfide-isomerase**

PFF score: **[125]**

Matched peptides No.: **[2]**  Sequence coverage %: **[5]** Matched sequences: **K.AFGAHAGNLNLK.A**

**K.YEDLASQFAASEFK.D**

Calculated Mr: **55623** Calculated *p*I: **4.76**

Probability Based Mowse Score:


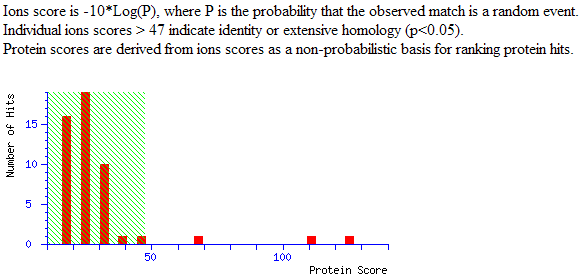


Matched peptide sequences: shown in Bold Red:


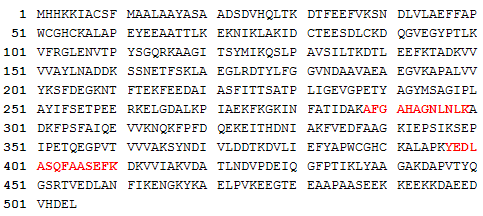


Spot No.:**35**

NCBI accession No.: **gi|342875717**

Species: ***Fusarium oxysporum* Fo5176**

Protein name: **UDP-N-acetylglucosamine pyrophosphorylase**

PFF score: **[402]**

Matched peptides No.: **[8]**  Sequence coverage %: **[29]** Matched sequences: **K.AGQEQVFTFYESLSSAER.G**

**R.GTLYQQLSGFDPAHINEITHR.A**

**K.TSDEPDRLEPLPESATASILDSSADDISK.W**

**K.SLFQLQGER.I**

**K.SAGSSPVVPWYVMTSGPTR.G + Oxidation (M)**

**K.FFQENNYFGLSQDNVK.I**

**K.VAVAPDGNGGLYNALVVSGVVDDMR.K**

**K.GTEITPPTLLWR.E**

Calculated Mr:**54902**  Calculated *p*I:**5.41**

Probability Based Mowse Score:


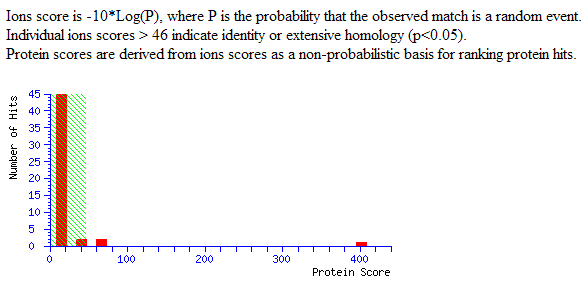


Matched peptide sequences: shown in Bold Red:


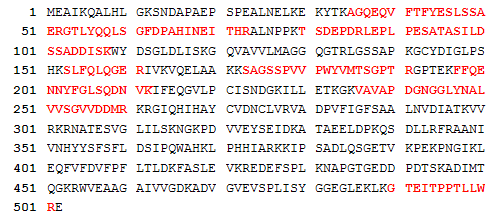


**Proteins identified in mycelium of R2**

Spot No.:**36**

NCBI accession No.: **gi|342872822**

Species: ***Fusarium oxysporum* Fo5176**

Protein name: **Hypothetical protein FOXB_14371**

PFF score: **[586]**

Matched peptides No.: **[6]**  Sequence coverage %: **[73]** Matched sequences: **M.GWFDNNTEVVENFNEYNQNSENR.E**

**K.LSHEIIGGAAAYEAAK.A**

**R.NGKPDSHAQAK.E**

**K.EFIAGAVGAFVDR.E**

**K.EFIAGAVGAFVDREFETK.G**

**K.GLDFFDREEAK.R**

Calculated Mr: **12416** Calculated *p*I: **5.21**

Probability Based Mowse Score:


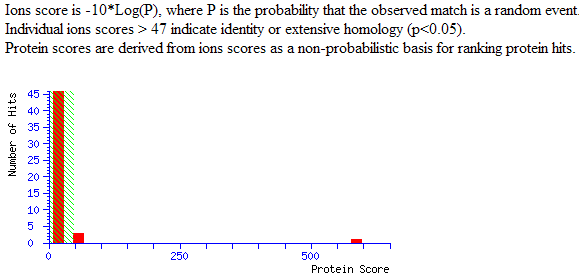


Matched peptide sequences: shown in Bold Red:


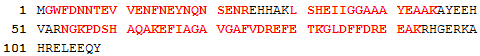


Spot No.: **37**

NCBI accession No.: **gi|342879727**

Species: ***Fusarium oxysporum* Fo5176**

Protein name: **Putative translation initiation inhibitor protein**

PFF score: **[108]**

Matched peptides No.: **[2]**  Sequence coverage %: **[17]** Matched sequences: **K.DAPAALGPYSQAIK.T**

**K.WFSHKPAR.S**

Calculated Mr: **13805** Calculated *p*I: **5.35**

Probability Based Mowse Score:


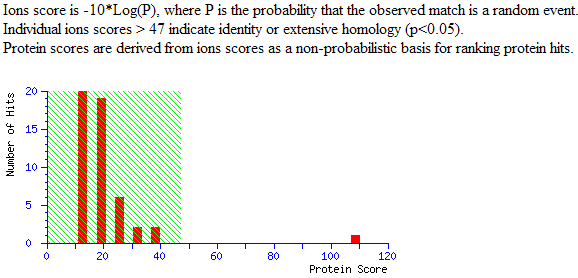


Matched peptide sequences: shown in Bold Red:


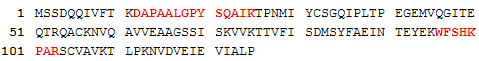


Spot No.: **38**

NCBI accession No.: **gi|342888991**

Species: ***Fusarium oxysporum* Fo5176**

Protein name: **Flavoprotein C26F1.14C**

PFF score: **[819]**

Matched peptides No.: **[10]**  Sequence coverage %: **[29]** Matched sequences: **R.ITCPWHGACFNAK.T**

**K.TGDIEDAPALDHLPVFQVAER.D**

**K.CAEFGSVQDDHVVVVGGGSGTLGVVESLR.E**

**K.GGITVISNEGYYPIDR.T**

**R.NKDFYEGGSIK.F**

**K.FVQGEVNNIDFSGR.F**

**K.NLGNIFTLR.N**

**K.DVYAIGDIATFPYHGPGGEGK.H**

**R.IEHWNVAQK.A**

**K.TEHFIPIFWSALGAQLR.Y**

Calculated Mr: **57855** Calculated *p*I: **6.13**

Probability Based Mowse Score:


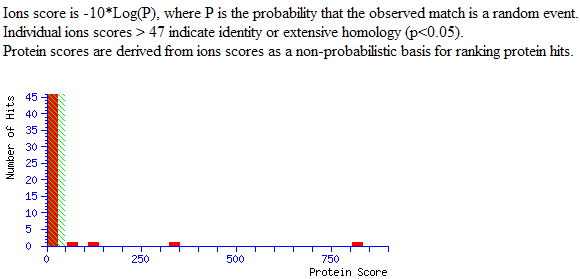


Matched peptide sequences: shown in Bold Red:


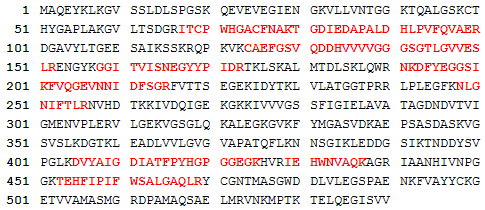


Spot No.: **39**

NCBI accession No.: **gi|116196014**

Species: ***Chaetomium globosum* CBS 148.51**

Protein name: **Probable hsp70-like protein**

PFF score: **[290]**

Matched peptides No.: **[4]**  Sequence coverage %: **[12]** Matched sequences: **K.DLLLLDVTPLSLGIETLGGVFTR.L**

**K.SQVFSTAADFQTAVEIK.V**

**K.LLGNFQLVGIPPAHR.G**

**R.GVPQVEVTFDIDADSIVHVHAK.D**

Calculated Mr: **68195** Calculated *p*I: **5.57**

Probability Based Mowse Score:


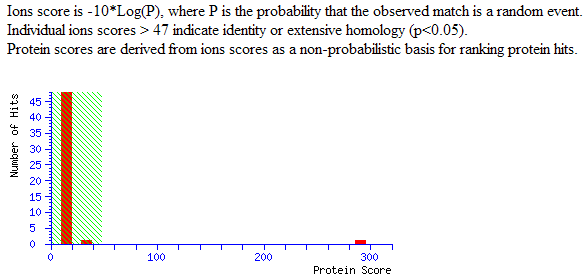


Matched peptide sequences: shown in Bold Red:


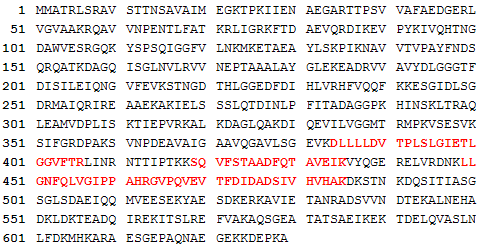


Spot No.: **40**

NCBI accession No.: **gi|342878854**

Species: ***Fusarium oxysporum* Fo5176**

Protein name: **Glucose-6-phosphate 1-epimerase**

PFF score: **[249]**

Matched peptides No.: **[4]** Sequence coverage %: **[23]** Matched sequences: **K.LWVSESAALDGSAPVR.G**

**R.GGIPIVFPVFGTAPDHEPVAK.L**

**K.GPSHPVVISESGTPR.F**

**R.DNLDQVVVWNPWVDK.S**

Calculated Mr: **31128** Calculated *p*I: **4.91**

Probability Based Mowse Score:


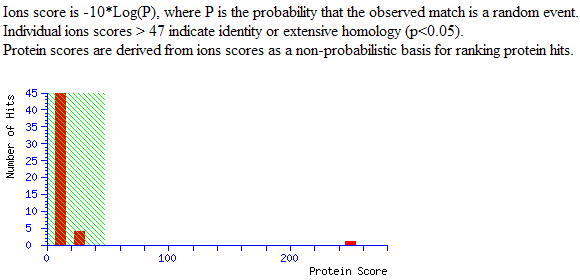


Matched peptide sequences: shown in Bold Red:


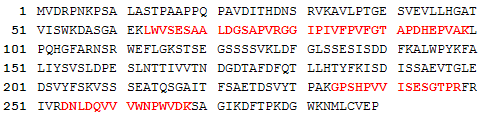


Spot No.: **41**

NCBI accession No.: **gi|342879240**

Species: ***Fusarium oxysporum* Fo5176**

Protein name: **Transaldolase**

PFF score: **[317]**

Matched peptides No.: **[5]**  Sequence coverage %: **[16]** Matched sequences: **K.YKPQDATTNPSLILAASK.K**

**K.ALHLIELYGEQGISK.D**

**K.YGYNTIVMGASFR.N**

**K.YGYNTIVMGASFR.N + Oxidation (M)**

**K.SYINDEALFR.F**

Calculated Mr: **36821** Calculated *p*I: **5.29**

Probability Based Mowse Score:


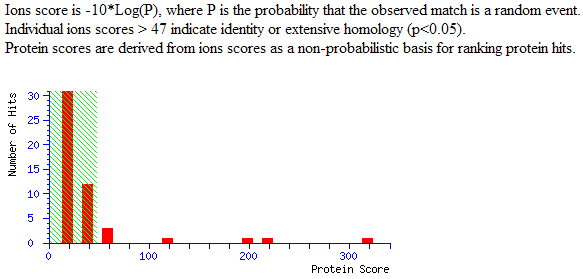


Matched peptide sequences: shown in Bold Red:


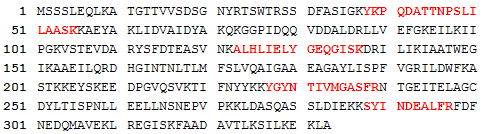


Spot No.: **42**

NCBI accession No.: **gi|342869347**

Species: ***Fusarium oxysporum* Fo5176**

Protein name: **Predicted hydrolases**

PFF score: **[224]**

Matched peptides No.: **[3]**  Sequence coverage %: **[16]** Matched sequences: **K.LPSVGSESHGVTWEADK.A**

**K.IIETAEPFFNQGR.E**

**R.LAAGHSPFLEK.T**

Calculated Mr: **27901** Calculated *p*I: **4.94**

Probability Based Mowse Score:


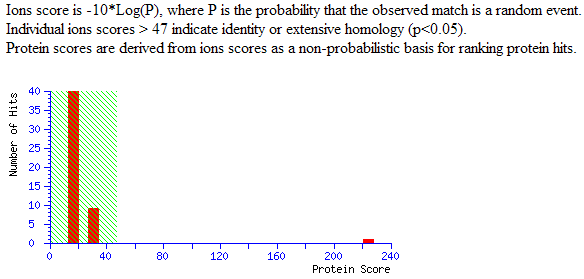


Matched peptide sequences: shown in Bold Red:


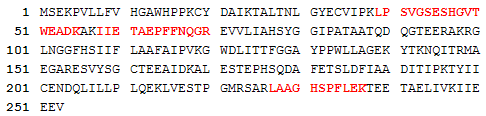


Spot No.: **43**

NCBI accession No.: **gi|110611696** 

Species: ***Fusarium oxysporum* f. sp. *lycopersici***

Protein name: **Putative pyruvate decarboxylase**

PFF score: **[151]**

Matched peptides No.: **[3]** Sequence coverage %: **[9]** Matched sequences: **K.LNKPSEIAEQIDTALR.T**

**K.GAVNEDHPNYGGVFAGDGSHPAR.A**

**K.NTDDSEIITQAWLWPR.V**

Calculated Mr: **63493** Calculated *p*I:**5.72**

Probability Based Mowse Score:


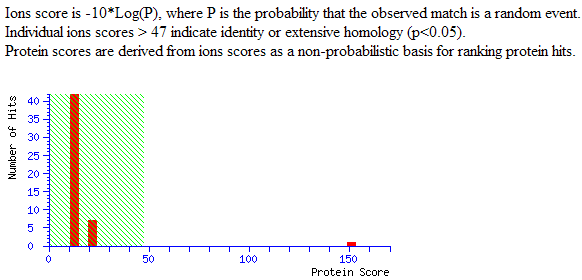


Matched peptide sequences: shown in Bold Red:


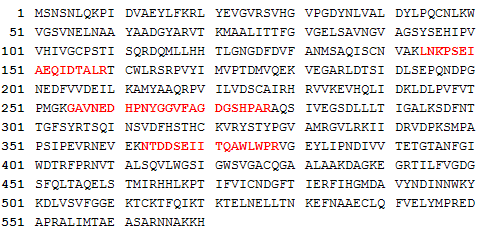


Spot No.: **44**

NCBI accession No.: **gi|342876993**

Species: ***Fusarium oxysporum* Fo5176**

Protein name: **Ketoreductases**

PFF score: **[292]**

Matched peptides No.: **[5]**  Sequence coverage %: **[10]** Matched sequences: **R.AYFYDGLPGDQR.L**

**K.SFFAEHLHEDEEIR.Y**

**R.SKDDDWVR.V**

**K.DDLLILPPGIYHR.F**

**R.FTTDESNYVHAMR.L**

Calculated Mr: **67042** Calculated *p*I: **5.45**

Probability Based Mowse Score:


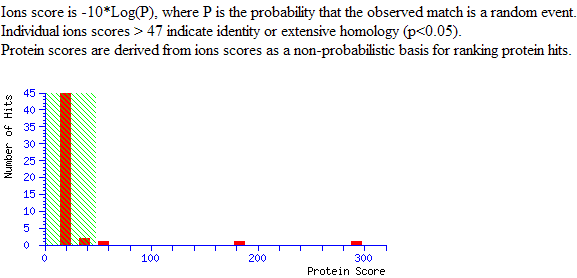


Matched peptide sequences: shown in Bold Red:


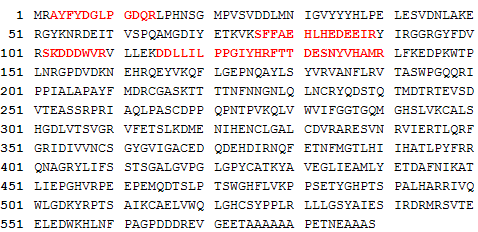


Spot No.: **45**

NCBI accession No.: **gi|46135811**

Species: ***Gibberella zeae* PH-1**

Protein name: **Septin like protein spn3**

PFF score: **[204]**

Matched peptides No.: **[3]**  Sequence coverage %: **[10]** Matched sequences: **K.IKPITVELELDEEGTR.I**

**R.HYPWGVVEVDNPR.H**

**K.EITHDFLYENYR.T**

Calculated Mr: **46553**  Calculated *p*I: **5.18**

Probability Based Mowse Score:


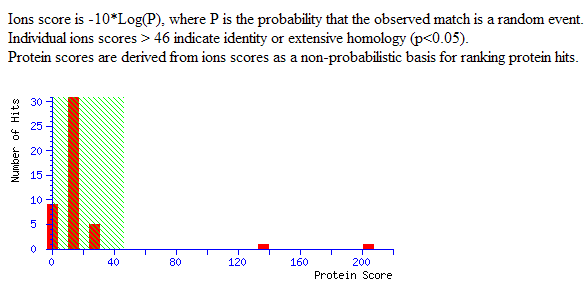


Matched peptide sequences: shown in Bold Red:


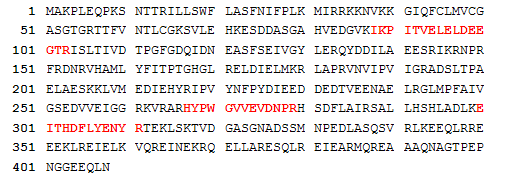


Spot No.: **46**

NCBI accession No.: **gi|342878833**

Species: ***Fusarium oxysporum* Fo5176**

Protein name: **CYB2-lactate dehydrogenase cytochrome b2**

PFF score: **[134]**

Matched peptides No.: **[4]** Sequence coverage %: **[15]** Matched sequences: **R.NNLEVFQR.Y**

**K.AGAAAIVFTVDSAADGNR.H**

**K.LAVQHGAPAIILSNHGGR.Q**

**K.HEIAIDAGNLGVPDVQK.I**

Calculated Mr: **42032**  Calculated *p*I: **6.03**

Probability Based Mowse Score:


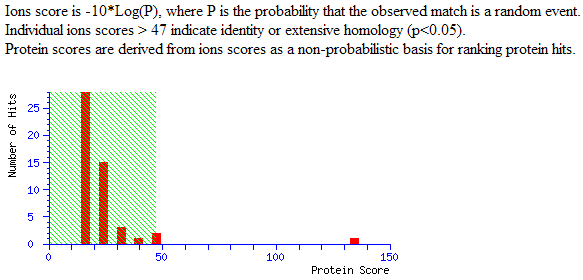


Matched peptide sequences: shown in Bold Red:


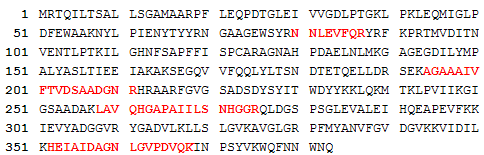


Spot No.: **47**

NCBI accession No.: **gi|110611696**

Species: ***Fusarium oxysporum* f. sp. *Lycopersici***

Protein name: **Putative pyruvate decarboxylase**

PFF score: **[359]**

Matched peptides No.: **[5]**  Sequence coverage %: **[14]** Matched sequences: **K.WVGSVNELNAAYAADGYAR.V**

**K.LNKPSEIAEQIDTALR.T**

**K.GAVNEDHPNYGGVFAGDGSHPAR.A**

**K.SDFNTTGFSYR.T**

**K.NTDDSEIITQAWLWPR.V**

Calculated Mr: **63493** Calculated *p*I: **5.72**

Probability Based Mowse Score:


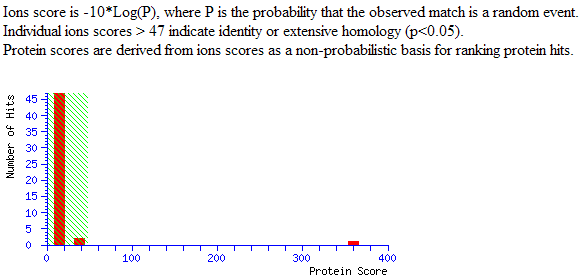


Matched peptide sequences: shown in Bold Red:


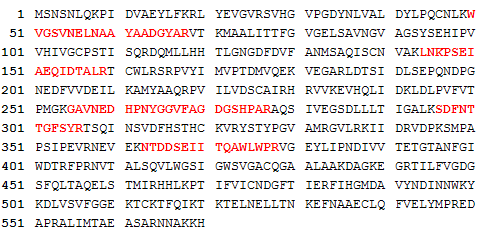


Spot No.: **48**

NCBI accession No.: **gi|189188250**

Species: ***Pyrenophora tritici-repentis* Pt-1C-BFP**

Protein name: **Cryptochrome-2**

PMF Mascot score:**82** Sequence coverage %:**30**

Matched peptides No.:**16** Total peptides No.:**30**

Calculated Mr: **74904** Calculated *p*I: **7.59**

Probability Based Mowse Score:


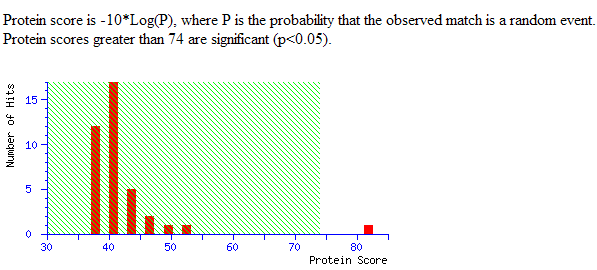


Matched peptide sequences: shown in Bold Red:


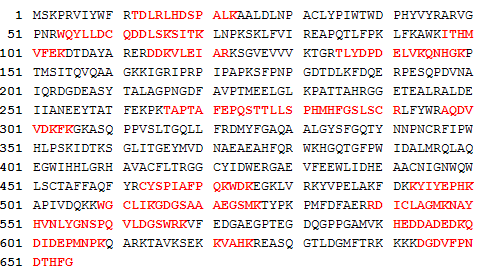


Spot No.: **49**

NCBI accession No.: **gi|342885951**

Species: ***Fusarium oxysporum* Fo5176**

Protein name: **Manganese superoxide dismutase precursor (sod-2)**

PFF score: **[474]**

Matched peptides No.: **[7]**  Sequence coverage %: **[40]** Matched sequences: **K.HHQAYVTNLNAALK.N**

**K.NYATATSTNDIAGQIALQSAIK.F**

**K.FNGGGHINHSLFWENLSPSSSADAK.P**

**K.TWGSIQAFQEAFK.K**

**K.TWGSIQAFQEAFKK.T**

**K.TLLGLQGSGWGWLVK.D**

**K.AAYVDNIWK.V**

Calculated Mr: **26531** Calculated *p*I: **6.22**

Probability Based Mowse Score:


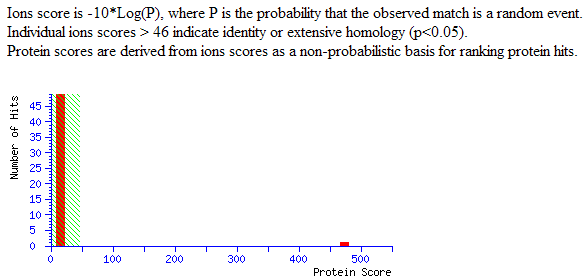


Matched peptide sequences: shown in Bold Red:


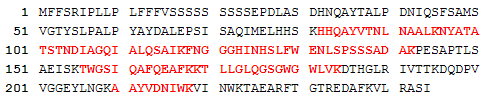


Spot No.: **50**

NCBI accession No.: **gi|342885052**

Species: ***Fusarium oxysporum* Fo5176**

Protein name: **ATP synthase subunit beta, mitochondrial**

PFF score: **[621]**

Matched peptides No.: **[9]**  Sequence coverage %: **[26]** Matched sequences: **K.IHQVIGAVVDVK.F**

**K.LVLEVSQHLGENVVR.C**

**R.LPIHTEAPEFVEQSTSAEVLVTGIK.V**

**K.VVDLLAPYAR.G**

**K.TVFIQELINNIAK.A**

**K.AHGGYSVFTGVGER.T**

**K.VALVFGQMNEPPGAR.A**

**R.IPSAVGYQPTLAVDMGGMQER.I**

**R.VVGQEHYDVATR.V**

Calculated Mr: **55170** Calculated *p*I: **5.27**

Probability Based Mowse Score:

Matched peptide sequences: shown in Bold Red:

Spot No.: **51**

NCBI accession No.: **gi|342882002**

Species: ***Fusarium oxysporum* Fo5176**

Protein name: **Thioredoxin-like protein**

PFF score: **[397]**

Matched peptides No.: **[5]**  Sequence coverage %: **[42]** Matched sequences: **K.ANPAWASSLTFIFR.Q**

**R.QQIQPWHPSSTLMHEAALAVLR.L + Oxidation (M)**

**K.VFELLVISDKPGEDGALNAGNK.V**

**R.LIGVHVTPTAVFDGVVQDVSSGWTK.E**

**K.EQWIEWLQK.N**

Calculated Mr: **24312** Calculated *p*I: **6.30**

Probability Based Mowse Score:

Matched peptide sequences: shown in Bold Red:

Spot No.: **52**

NCBI accession No.: **gi|342877147**

Species: ***Fusarium oxysporum* Fo5176**

Protein name: **Dihydrolipoyl dehydrogenase precursor**

PFF score: **[468]**

Matched peptides No.: **[9]**  Sequence coverage %: **[26]** Matched sequences: **K.DLVVIGGGVAGYVAAIK.A**

**R.GSLGGTCLNVGCIPSK.S**

**K.GAGSFVNEHEIK.V**

**K.GGKPESIESDVVLVAIGR.R**

**R.RPYTGGLGLENIGLEADDR.G**

**R.CVGDVTFGPMLAHK.A + Oxidation (M)**

**K.AEEEAVAVVEYIK.K**

**R.VGTFPFVANSR.A**

**R.TCHAHPTLAEAFK.E**

Calculated Mr: **54516** Calculated *p*I: **6.70**

Probability Based Mowse Score:

Matched peptide sequences: shown in Bold Red:

Spot No.: **53**

NCBI accession No.: **gi|302910270**

Species: ***Nectria haematococca* mpVI 77-13-4**

Protein name: **Glucose-6-phosphate isomerase**

PFF score: **[161]**

Matched peptides No.: **[3]**  Sequence coverage %: **[5]** Matched sequences: **M.APANTLPAWSDLQAHR.D**

**K.NMFGFESWVGGR.Y**

**K.NMFGFESWVGGR.Y + Oxidation (M)**

Calculated Mr: **60870**  Calculated *p*I: **5.42**

Probability Based Mowse Score:

Matched peptide sequences: shown in Bold Red:

Spot No.: **54**

NCBI accession No.: **gi|342888046**

Species: ***Fusarium oxysporum* Fo5176**

Protein name: **UDP-glucose 4-epimerase Gal10**

PFF score: **[560]**

Matched peptides No.: **[8]**  Sequence coverage %: **[13]** Matched sequences: **R.VNVGGSISLLR.S**

**R.NNVNNIVFSSSATVYGDATR.F**

**R.FPNMIPIPEHCPIGPTNTYGR.T + Oxidation (M)**

**K.SMIEDVITDFINAQR.S + Oxidation (M)**

**K.LLVFGDDYSSR.D**

**R.DYIHVVDLAR.G**

**R.DLPYEVRPR.R**

**R.RQGDVLDLTANPTLANK.E**

Calculated Mr: **40378** Calculated *p*I: **5.65**

Probability Based Mowse Score:

Matched peptide sequences: shown in Bold Red:

Spot No.: **55**

NCBI accession No.: **gi|342886297**

Species: ***Fusarium oxysporum* Fo5176**

Protein name: **Heat shock protein 60, mitochondrial**

PFF score: **[399]**

Matched peptides No.: **[9]**  Sequence coverage %: **[12]** Matched sequences: **R.GIQAAVEAVVEFLQK.N**

**K.TVADELEVTEGMR.F**

**K.VEFENPLILLSEK.K**

**K.KISAVQDIIPALEVSTQQR.R**

**K.ISAVQDIIPALEVSTQQR.R**

**R.RPLVIIAEDIEGEALAVCILNK.L**

**K.SILGDLAILTDGTVFTDELDIK.L**

**R.FVDALNATR.A**

**K.ASAHALNEVPTANFDQQLGVSIVK.N**

Calculated Mr: **27901** Calculated *p*I: **4.94**

Probability Based Mowse Score:

Matched peptide sequences: shown in Bold Red:

Spot No.: **56**

NCBI accession No.: **gi|342885916**

Species: ***Fusarium oxysporum* Fo5176**

Protein name: **TPI1-triose-phosphate isomerase**

PFF score: **[197]**

Matched peptides No.: **[3]**  Sequence coverage %: **[17]** Matched sequences: **K.NAEVVVSPPAIYLPLVR.E**

**K.DSDINWVILGHSER.R**

**K.VIWCCGETLETR.E**

Calculated Mr: **27245** Calculated *p*I: **5.29**

Probability Based Mowse Score:

Matched peptide sequences: shown in Bold Red:

Spot No.: **57**

NCBI accession No.: **gi|342888721**

Species: ***Fusarium oxysporum* Fo5176**

Protein name: **Members of the aldo/keto reductase family**

PFF score: **[295]**

Matched peptides No.: **[6]**  Sequence coverage %: **[20]** Matched sequences: **K.NKTELDQGVTLSQTWEAVTK.L**

**K.TELDQGVTLSQTWEAVTK.L**

**R.SIGVSNFSIEMLETIIK.D + Oxidation (M)**

**K.DTGVTPAINQVER.H**

**K.TYSPDWDIDVFGDEK.E**

**K.TYSPDWDIDVFGDEKEK.T**

Calculated Mr: **37413** Calculated *p*I: **5.91**

Probability Based Mowse Score:

Matched peptide sequences: shown in Bold Red:

Spot No.: **58**

NCBI accession No.: **gi|70997019**

Species: ***Aspergillus fumigatus* Af293**

Protein name: **Hsp90 binding co-chaperone (Sba1)**

PFF score: **[63]**

Matched peptides No.: **[1]** Sequence coverage %: **[4]** Matched sequences: **K.ELKEEYWPR.L**

Calculated Mr: **22199** Calculated *p*I: **4.36**

Probability Based Mowse Score:

Matched peptide sequences: shown in Bold Red:

Spot No.: **59**

NCBI accession No.: **gi|46117136**

Species: ***Gibberella zeae* PH-1**

Protein name: **PSA2_NEUCR Probable proteasome subunit alpha type 2**

PFF score: **[165]**

Matched peptides No.: **[4]**  Sequence coverage %: **[13]** Matched sequences: **R.YSFSLTTFSPSGK.L**

**K.RIYNEYPPTR.I**

**R.IYNEYPPTR.I**

**K.GGPMLYQVDPSGSYYPWK.A + Oxidation (M)**

Calculated Mr: **30543** Calculated *p*I: **4.99**

Probability Based Mowse Score:

Matched peptide sequences: shown in Bold Red:

Spot No.: **60**

NCBI accession No.: **gi|342886090**

Species: ***Fusarium oxysporum* Fo5176**

Protein name: **Arginase**

PFF score: **[657]**

Matched peptides No.: **[7]**  Sequence coverage %: **[29]** Matched sequences: **R.FLSKPDEVGVVAVGFSGGQPK.A**

**K.AGVDIGPAALIESGLLTEIR.D**

**K.LFGDESVQQFEDLIPSSDPDYR.G**

**K.IASHTYEHSR.E**

**K.EDNEEYFGWLEDDMR.L**

**K.AFSMHDVDR.H**

**K.AFSMHDVDR.H + Oxidation (M)**

Calculated Mr:**35333**  Calculated *p*I: **5.23**

Probability Based Mowse Score:

Matched peptide sequences: shown in Bold Red:

Spot No.: **61**

NCBI accession No.: **gi|342885951**

Species: ***Fusarium oxysporum* Fo5176**

Protein name: **Manganese superoxide dismutase precursor (sod-2)**

PFF score: **[213]**

Matched peptides No.: **[3]**  Sequence coverage %: **[20]** Matched sequences: **K.HHQAYVTNLNAALK.N**

**K.NYATATSTNDIAGQIALQSAIK.F**

**K.TWGSIQAFQEAFK.K**

Calculated Mr: **26531** Calculated *p*I: **6.22**

Probability Based Mowse Score:

Matched peptide sequences: shown in Bold Red:

Spot No.: **62**

NCBI accession No.: **gi|342874907**

Species: ***Fusarium oxysporum* Fo5176**

Protein name: **SOD2-superoxide dismutase (Mn) precursor, mitochondrial**

PFF score: **[206]**

Matched peptides No.: **[2]**  Sequence coverage %: **[13]** Matched sequences: **K.HHQTYVTGFNNATDALAEAQHK.N**

**K.EDFGSFDSFK.K**

Calculated Mr: **25122** Calculated *p*I: **7.96**

Probability Based Mowse Score:

Matched peptide sequences: shown in Bold Red:

Spot No.: **63**

NCBI accession No.: **gi|46108708**

Species: ***Gibberella zeae* PH-1**

Protein name: **20S proteasome, beta subunit**

PFF score: **[212]**

Matched peptides No.: **[3]**  Sequence coverage %: **[23]** Matched sequences: **R.TFANLVSSSLYER.R**

**R.FGPYFVSPVVAGLDPK.T**

**R.DALSGWGAHVYIIEK.D**

Calculated Mr: **21494** Calculated *p*I: **4.98**

Probability Based Mowse Score:

Matched peptide sequences: shown in Bold Red:

Spot No.: **64**

NCBI accession No.: **gi|120690**

Species: ***Cryphonectria parasitica***

Protein name: **Glyceraldehyde-3-phosphate dehydrogenase**

PFF score: **[101]**

Matched peptides No.: **[1]**  Sequence coverage %: **[3]** Matched sequences: **K.LVSWYDNEWGYSR.R**

Calculated Mr:**36285**  Calculated *p*I: **6.72**

Probability Based Mowse Score:

Matched peptide sequences: shown in Bold Red:

Spot No.: **65**

NCBI accession No.: **gi|342883238**

Species: ***Fusarium oxysporum* Fo5176**

Protein name: **Hypothetical protein FOXB_05715**

PFF score: **[412]**

Matched peptides No.: **[7]**  Sequence coverage %: **[35]** Matched sequences: **K.TADHGPIIESLKPIVDFSK.S**

**K.HVTLTPFPPEDVLNK.D**

**R.RLEEFTGVALK.T**

**R.GISYGWSVENDVPVR.G**

**R.GISYGWSVENDVPVRGDESK.T**

**R.ETDDFKENIGLLR.E**

**K.AHEHTHDHGGGCC.-**

Calculated Mr: **28725** Calculated *p*I: **5.01**

Probability Based Mowse Score:

Matched peptide sequences: shown in Bold Red:

Spot No.: **66**

NCBI accession No.: **gi|225877962**

Species: ***Gibberella fujikuroi***

Protein name: **Saccharopine dehydrogenase**

PFF score: **[201]**

Matched peptides No.: **[4]**  Sequence coverage %: **[13]** Matched sequences: **K.TLVDAGYPISVER.S**

**R.IFEDSEYEAAGAR.L**

**R.GYYLNEDELVNQIR.E**

**R.KPTALVLGALGR.C**

Calculated Mr: **43543** Calculated *p*I: **5.17**

Probability Based Mowse Score:

Matched peptide sequences: shown in Bold Red:

Spot No.: **67**

NCBI accession No.: **gi|342884543**

Species: ***Fusarium oxysporum* Fo5176**

Protein name: **Enolase**

PFF score: **[510]**

Matched peptides No.: **[6]**  Sequence coverage %: **[18]** Matched sequences: **R.GNPTVEVDVVTETGLHR.A**

**K.TQDIQIVGDDLTVTNPLR.I**

**K.DSYADGWGVMVSHR.S**

**K.DSYADGWGVMVSHR.S + Oxidation (M)**

**R.SGETEDVTIADIAVGLR.A**

**R.IEEELGDQAIYPGANFR.K**

Calculated Mr: **48508** Calculated *p*I: **5.17**

Probability Based Mowse Score:

Matched peptide sequences: shown in Bold Red:

Spot No.: **68**

NCBI accession No.: **gi|342865956**

Species: ***Fusarium oxysporum* Fo5176**

Protein name: **Adenosine kinase**

PFF score: **[443]**

Matched peptides No.: **[7]**  Sequence coverage %: **[31]** Matched sequences: **K.HLPLYEDLLNNYDAK.L**

**R.GAQYILPPNSVVYLGGAGDDK.Y**

**K.YAAILHDAVK.A**

**R.SLCTDLGAANHYDLDHLK.K**

**R.IAIVTQGTDPTLVAIQGEDDIK.E**

**K.EFPVHAIEK.E**

**R.LSIQELGPSYPFPK.Q**

Calculated Mr: **38124** Calculated *p*I: **5.42**

Probability Based Mowse Score:

Matched peptide sequences: shown in Bold Red:

**Proteins identified in conidia of R1**

Spot No.: **1**

NCBI accession No.: **gi|46135911**

Species: ***Gibberella zeae* PH-1**

Protein name: **GR78_NEUCR 78 KDA GLUCOSE-REGULATED PROTEIN HOMOLOG PRECURSOR (GRP 78)**

PFF score: **[117]**

Matched peptides No.: **[3]**  Sequence coverage %: **[5]** Matched sequences: **K.GKVEILVNDQGNR.I**

**R.ITPSYVAFTEDER.L**

**K.FELTGIPPAPR.G**

Calculated Mr: **74638** Calculated *p*I: **5.08**

Probability Based Mowse Score:

Matched peptide sequences: shown in Bold Red:

Spot No.: **2**

NCBI accession No.: **gi|46137663**

Species: ***Gibberella zeae* PH-1**

Protein name: **Isocitrate dehydrogenase [NADP], mitochondrial**

PFF score: **[87]**

Matched peptides No.: **[3]**  Sequence coverage %: **[8]** Matched sequences: **K.YYDLGLEYR.D**

**K.TFESEAAHGTVTR.H**

**K.GNETSTNPIASIFAWTR.G**

Calculated Mr: **50196** Calculated *p*I: **7.07**

Probability Based Mowse Score:

Matched peptide sequences: shown in Bold Red:

Spot No.: **3**

NCBI accession No.: **gi|342879255**

Species: ***Fusarium oxysporum* Fo5176**

Protein name: **Hypothetical protein FOXB_08970**

PFF score: **[179]**

Matched peptides No.: **[4]**  Sequence coverage %: **[5]** Matched sequences: **R.DIEIDIHEGPR.P**

**R.LGDFLMLQGR.P**

**R.LGDFLMLQGR.P + Oxidation (M)**

**R.YLGVDLFTK.Q**

Calculated Mr: **66373** Calculated *p*I: **6.50**

Probability Based Mowse Score:

Matched peptide sequences: shown in Bold Red:

Spot No.:**4**

NCBI accession No.:**gi|342875528**

Species: ***Fusarium oxysporum* Fo5176**

Protein name: **Hsp90 associated co-chaperone**

PFF score: **[336]**

Matched peptides No.: **[4]**  Sequence coverage %: **[24]** Matched sequences:**K.NFIYLTISVPDVPKDSLTLDLQPTK.L**

**K.KYHVELEFWGEIDPAESK.I**

**K.YHVELEFWGEIDPAESK.I**

**K.ELKEEYWPR.L**

Calculated Mr: **23030**  Calculated *p*I: **4.40**

Probability Based Mowse Score:

Matched peptide sequences: shown in Bold Red:

Spot No.: **5**

NCBI accession No.: **gi|342881721**

Species: ***Fusarium oxysporum* Fo5176**

Protein name: **DJ-1/PfpI family protein**

PFF score: **[176]**

Matched peptides No.: **[3]**  Sequence coverage %: **[22]** Matched sequences: **K.AGKPVAAVCHGPIVFVNVK.V**

**K.VDGKPLLEGR.E K.LAVDGQVITGQNPASAHAVGK.A**

Calculated Mr: **24474** Calculated *p*I: **5.12**

Probability Based Mowse Score:

Matched peptide sequences: shown in Bold Red:

Spot No.: **6**

NCBI accession No.: **gi|342890490**

Species: ***Fusarium oxysporum* Fo5176**

Protein name: **Hypothetical protein FOXB_00261**

PMF Mascot score: **78** Sequence coverage %: **38** Matched peptides No.: **7** Total peptides No.: **59**

Calculated Mr: **16478** Calculated *p*I:**5.05**

Probability Based Mowse Score:

Matched peptide sequences: shown in Bold Red:

Spot No.: **7**

NCBI accession No.: **gi|342888423**

Species: ***Fusarium oxysporum* Fo5176**

Protein name: **Fo5176-SIX1**

PFF score: **[182]**

Matched peptides No.: **[2]**  Sequence coverage %: **[10]** Matched sequences: **R.HTEWEGTWYPESPK.S**

**R.GNGHSWACINCPGGK.L**

Calculated Mr: **31302** Calculated *p*I: **8.54**

Probability Based Mowse Score:

Matched peptide sequences: shown in Bold Red:

Spot No.: **8**

NCBI accession No.: **gi|342885052**

Species: ***Fusarium oxysporum* Fo5176**

PFF score: **[139]**

Protein name: **ATP synthase subunit beta, mitochondrial**

Matched peptides No.: **[5]**  Sequence coverage %: **[9]** Matched sequences: **K.VVDLLAPYAR.G**

**K.AHGGYSVFTGVGER.T**

**K.VALVFGQMNEPPGAR.A**

**K.VALVFGQMNEPPGAR.A + Oxidation (M)**

**R.VVGQEHYDVATR.V**

Calculated Mr: **55170** Calculated *p*I: **5.27**

Probability Based Mowse Score:

Matched peptide sequences: shown in Bold Red:

Spot No.: **9**

NCBI accession No.: **gi|336378323**

Species: ***Serpula lacrymans* var. *lacrymans* S7.9**

Protein name: **Nucleoporin-interacting protein NIC96**

PMF Mascot score: **78** Sequence coverage %: **16** Matched peptides No.: **17** Total peptides No.: **82**

Calculated Mr: **96461** Calculated *p*I:**6.72**

Probability Based Mowse Score:

Matched peptide sequences: shown in Bold Red:

Spot No.: **10**

NCBI accession No.: **gi|342874660**

Species: ***Fusarium oxysporum* Fo5176**

Protein name: **Isovaleryl-CoA dehydrogenase**

PFF score: **[305]**

Matched peptides No.: **[4]**  Sequence coverage %: **[11]** Matched sequences: **K.GFEAPSNEELEELR.E**

**R.VLMEGLDLER.L**

**R.TQDCAGAILYAAER.A**

**K.LYEIGAGTSEIR.R**

Calculated Mr: **46467** Calculated *p*I: **7.02**

Probability Based Mowse Score:

Matched peptide sequences: shown in Bold Red:

Spot No.:**11**

NCBI accession No.: **gi|342881938**

Species: ***Fusarium oxysporum* Fo5176**

Protein name: **Hypothetical protein FOXB_06772**

PFF score: **[145]**

Matched peptides No.: **[3]**  Sequence coverage %: **[16]** Matched sequences: **K.DCLSLWADANVCVR.T**

**K.FIFEIYNEWGIR.Q**

**K.SWHTETYLEK.G**

Calculated Mr: **24153**  Calculated *p*I:**5.54**

Probability Based Mowse Score:

Matched peptide sequences: shown in Bold Red:

Spot No.: **12**

NCBI accession No.: **gi|342874407**

Species: ***Fusarium oxysporum* Fo5176**

Protein name: **NADH-ubiquinone oxidoreductase 17.8 kDa subunit**

PFF score: **[126]**

Matched peptides No.: **[3]**  Sequence coverage %: **[26]** Matched sequences:

**R.FVDVAYPEAMTSYAPR.N + Oxidation (M)**

**R.NNVAGHLANMDYVVEHYR.Q + Oxidation (M)**

**R.QKHIQEEER.K**

Calculated Mr: **18589** Calculated *p*I: **6.71**

Probability Based Mowse Score:

Matched peptide sequences: shown in Bold Red:

Spot No.:**13**

NCBI accession No.: **gi|342876133**

Species: ***Fusarium oxysporum* Fo5176**

Protein name: **Hypothetical protein FOXB_11655**

PFF score: **[567]**

Matched peptides No.: **[5]**  Sequence coverage %: **[44]** Matched sequences: **K.TLSDSSEPILSLQGIGYLTR.K**

**R.EHTDWLFGTVR.G**

**R.FVTLDEIEEDFLK.T**

**K.TGWLVEGDGK.F**

**K.FIQSIAESVDNGWVANQIWGFEEINGER.R**

Calculated Mr: **20881**  Calculated *p*I:**5.39**

Probability Based Mowse Score:

Matched peptide sequences: shown in Bold Red:

Spot No.: **14**

NCBI accession No.: **gi|46135911**

Species: ***Gibberella zeae* PH-1**

Protein name: **GR78_NEUCR 78 KDA GLUCOSE-REGULATED PROTEIN HOMOLOG PRECURSOR (GRP 78)**

PFF score: **[173]**

Matched peptides No.: **[5]**  Sequence coverage %: **[8]** Matched sequences: **K.GKVEILVNDQGNR.I**

**R.ITPSYVAFTEDER.L**

**K.KVTHAVVTVPAYFNDNQR.Q**

**K.VTHAVVTVPAYFNDNQR.Q**

**K.DAGIIAGLNVLR.I**

Calculated Mr: **74638** Calculated *p*I: **5.08**

Probability Based Mowse Score:

Matched peptide sequences: shown in Bold Red:

Spot No.:**15**

NCBI accession No.: **gi|342888423**

Species: ***Fusarium oxysporum* Fo5176**

Protein name: **Fo5176-SIX1**

PFF score: **[65]**

Matched peptides No.: **[1]** Sequence coverage %: **[5]** Matched sequences:**R.HTEWEGTWYPESPK.S**

Calculated Mr: **31302**  Calculated *p*I:**8.54**

Probability Based Mowse Score:

Matched peptide sequences: shown in Bold Red:

Spot No.: **16**

NCBI accession No.: **gi|46128719**

Species: ***Gibberella zeae* PH-1**

Protein name: **Hypothetical protein FG08737.1**

PFF score: **[87]**

Matched peptides No.: **[2]** Sequence coverage %: **[10]** Matched sequences: **R.LGDFLMLQGR.P**

**K.QGLPVIDQSNLYSR.L**

Calculated Mr: **24959** Calculated *p*I: **5.77**

Probability Based Mowse Score:

Matched peptide sequences: shown in Bold Red:

Spot No.: **17**

NCBI accession No.: **gi|168256**

Species: ***Ajellomyces capsulatus***

Protein name: **Heat shock protein 82**

PFF score: **[125]**

Matched peptides No.: **[2]**  Sequence coverage %: **[3]** Matched sequences: **K.HFSVEGQLEFR.A**

**K.RAPFDLFETK.K**

Calculated Mr: **77211** Calculated *p*I: **4.87**

Probability Based Mowse Score:

Matched peptide sequences: shown in Bold Red:

Spot No.: **18**

NCBI accession No.: **gi|342888423**

Species: ***Fusarium oxysporum* Fo5176**

Protein name: **Fo5176-SIX1**

PFF score: **[86]**

Matched peptides No.: [1] Sequence coverage %: [5] Matched sequences: ***R.GNGHSWACINCPGGK.L***

Calculated Mr: **31302** Calculated *p*I: **8.54**

Probability Based Mowse Score:

Matched peptide sequences: shown in Bold Red:

Spot No.: **19**

NCBI accession No.: **gi|342888423**

Species: ***Fusarium oxysporum* Fo5176**

Protein name: **Fo5176-SIX1**

PFF score: **[58]**

Matched peptides No.: **[1]**  Sequence coverage %: **[5]** Matched sequences: **R.GNGHSWACINCPGGK.L**

Calculated Mr: **31302** Calculated *p*I: **8.54**

Probability Based Mowse Score:

Matched peptide sequences: shown in Bold Red:

Spot No.:**20**

NCBI accession No.: **gi|62275449**

Species: ***Fusarium oxysporum***

Protein name: **Beta-1,3-glucanosyltransferase**

PFF score: **[225]**

Matched peptides No.: **[3]**  Sequence coverage %: **[7]** Matched sequences: **K.WDVELYER.Y**

**R.WLGVGYASNDDVDIR.E**

**R.EQIADYFNCGDDDSR.I**

Calculated Mr: **59047** Calculated *p*I: **4.83**

Probability Based Mowse Score:

Matched peptide sequences: shown in Bold Red:

**Proteins identified in conidia of R2**

Spot No.: **21**

NCBI accession No.: **gi|302687328**

Species: ***Schizophyllum commune* H4-8**

Protein name: **40S ribosomal protein S4**

PMF Mascot score:**77**  Sequence coverage %: **50**

Matched peptides No.:**14** Total peptides No.:**48**

Calculated Mr: **29700** Calculated *p*I: **10.29**

Probability Based Mowse Score:

Matched peptide sequences: shown in Bold Red:

Spot No.: **22**

NCBI accession No.: **gi|342877157**

Species: ***Fusarium oxysporum* Fo5176**

Protein name: **Pyruvate decarboxylase**

PMF Mascot score:**132**  Sequence coverage %: **62**

Matched peptides No.:**28** Total peptides No.:**59**

Calculated Mr: **63503**  Calculated *p*I:**5.72**

Probability Based Mowse Score:

Matched peptide sequences: shown in Bold Red:

Spot No.: **23**

NCBI accession No.: **gi|342877850**

Species: ***Fusarium oxysporum* Fo5176**

Protein name: **Glu/asp-tRNA amidotransferase subunit A**

PMF Mascot score:**76**  Sequence coverage %: **37**

Matched peptides No.:**18** Total peptides No.:**46**

Calculated Mr: **63631** Calculated *p*I: **6.86**

Probability Based Mowse Score:

Matched peptide sequences: shown in Bold Red:

Spot No.: **24**

NCBI accession No.: **gi|342869186**

Species: ***Fusarium oxysporum* Fo5176**

PFF score: **[201]**

Protein name: **Hypothetical protein FOXB_16439**

Matched peptides No.: **[2]**  Sequence coverage %: **[12]** Matched sequences: **R.DIACGPSTPPREDNIGTK.G**

**R.LQEDIAGASVSGHVR.S**

Calculated Mr: **29553** Calculated *p*I: **5.59**

Probability Based Mowse Score:

Matched peptide sequences: shown in Bold Red:

Spot No.: **25**

NCBI accession No.: **gi|342885916**

Species: ***Fusarium oxysporum* Fo5176**

Protein name: **TPI1-triose-phosphate isomerase**

PMF Mascot score:**98**  Sequence coverage %: **64**

Matched peptides No.:**14** Total peptides No.:**76**

Calculated Mr: **27245** Calculated *p*I: **5.29**

Probability Based Mowse Score:

Matched peptide sequences: shown in Bold Red:

Spot No.:**26**

NCBI accession No.: **gi|342879371**

Species: ***Fusarium oxysporum* Fo5176**

Protein name: **Inorganic pyrophosphatase**

PMF Mascot score:**159**  Sequence coverage %: **77**

Matched peptides No.:**19** Total peptides No.:**45**

Calculated Mr: **32863** Calculated *p*I: **5.13**

Probability Based Mowse Score:

Matched peptide sequences: shown in Bold Red:

Spot No.: **27**

NCBI accession No.: **gi|342871910**

Species: ***Fusarium oxysporum* Fo5176**

Protein name: **Fructose-bisphosphate aldolase**

PMF Mascot score:**103**  Sequence coverage %: **59**

Matched peptides No.:**16** Total peptides No.:**42**

Calculated Mr: **37389** Calculated *p*I: **5.24**

Probability Based Mowse Score:

Matched peptide sequences: shown in Bold Red:

Spot No.: **28**

NCBI accession No.: **gi|342871910**

Species: ***Fusarium oxysporum* Fo5176**

Protein name: **Fructose-bisphosphate aldolase**

PMF Mascot score:**87**  Sequence coverage %: **46**

Matched peptides No.:**12** Total peptides No.:**29**

Calculated Mr: **37389** Calculated *p*I: **5.24**

Probability Based Mowse Score:

Matched peptide sequences: shown in Bold Red:

Spot No.: **29**

NCBI accession No.: **gi|342875953**

Species: ***Fusarium oxysporum* Fo5176**

Protein name: **Glycosidase crf1**

PFF score: **[136]**

Matched peptides No.: **[3]**  Sequence coverage %: **[17]** Matched sequences:

**K.CNPMTATCPADPAFGR.D + Oxidation (M)**

**K.SISIVDYAGTDAPTTSSVR.E**

**R.EYLFGDHSGSWK.S**

Calculated Mr:**29591**  Calculated *p*I: **4.47**

Probability Based Mowse Score:

Matched peptide sequences: shown in Bold Red:

Spot No: **30**

NCBI accession No.: **gi|342875953**

Species: ***Fusarium oxysporum* Fo5176**

Protein name: **Glycosidase crf1**

PFF score: **[94]**

Matched peptides No.: **[2]**  Sequence coverage %: **[9]** Matched sequences: **K.SISIVDYAGTDAPTTSSVR.E**

**K.YIFFGR.V**

Calculated Mr: **29591** Calculated *p*I: **4.47**

Probability Based Mowse Score:

Matched peptide sequences: shown in Bold Red:

Spot No.: **31**

NCBI accession No.: **gi|302899157**

Species: ***Nectria haematococca* mpVI 77-13-4**

Protein name: **Hsp90 associated co-chaperone**

PFF score: **[46]**

Matched peptides No.: **[1]**  Sequence coverage %: **[8]** Matched sequences: **K.YHVELEFWGEIDPAESK.I**

Calculated Mr:**23370**  Calculated *p*I: **4.47**

Probability Based Mowse Score:

Matched peptide sequences: shown in Bold Red:

Spot No.: **32**

NCBI accession No.: **gi|342875953**

Species: ***Fusarium oxysporum* Fo5176**

Protein name: **Glycosidase crf1**

PFF score: **[175]**

Matched peptides No.: **[2]**  Sequence coverage %: **[11]** Matched sequences: **K.SISIVDYAGTDAPTTSSVR.E**

**R.EYLFGDHSGSWK.S**

Calculated Mr: **29591** Calculated *p*I: **4.47**

Probability Based Mowse Score:

Matched peptide sequences: shown in Bold Red:

Spot No.: **33**

NCBI accession No.: **gi|342875953**

Species: ***Fusarium oxysporum* Fo5176**

Protein name: **Glycosidase crf1**

PFF score: **[113]**

Matched peptides No.: **[2]**  Sequence coverage %: **[11]** Matched sequences: **K.SISIVDYAGTDAPTTSSVR.E**

**R.EYLFGDHSGSWK.S**

Calculated Mr: **29591** Calculated *p*I: **4.47**

Probability Based Mowse Score:

Matched peptide sequences: shown in Bold Red:

Spot No.: **34**

NCBI accession No.: **gi|342875953**

Species: ***Fusarium oxysporum* Fo5176**

Protein name: **Glycosidase crf1**

PFF score: **[165]**

Matched peptides No.: **[2]**  Sequence coverage %: **[11]** Matched sequences: **K.SISIVDYAGTDAPTTSSVR.E**

**R.EYLFGDHSGSWK.S**

Calculated Mr: **29591** Calculated *p*I: **4.47**

Probability Based Mowse Score:

Matched peptide sequences: shown in Bold Red:

Spot No.: **35**

NCBI accession No.: **gi|342885916**

Species: ***Fusarium oxysporum* Fo5176**

Protein name: **TPI1-triose-phosphate isomerase**

PFF score: **[241]**

Matched peptides No.: **[4]**  Sequence coverage %: **[19]** Matched sequences: **K.DSDINWVILGHSER.R**

**K.VIWCCGETLETR.E**

**K.VATTEQAQEVHK.A**

**R.ILYGGSVNEK.N**

Calculated Mr: **27245** Calculated *p*I: **5.29**

Probability Based Mowse Score:

Matched peptide sequences: shown in Bold Red:

Spot No.: **36**

NCBI accession No.: **gi|170091162**

Species: ***Laccaria bicolor* S238N-H82**

Protein name: **Serine/threonine protein kinases**

PMF Mascot score: **79**  Sequence coverage %: **73**

Matched peptides No.:**8** Total peptides No.:**68**

Calculated Mr: **13954** Calculated *p*I: **5.32**

Probability Based Mowse Score:

Matched peptide sequences: shown in Bold Red:

Spot No.: **37**

NCBI accession No.: **gi|342875953**

Species: ***Fusarium oxysporum* Fo5176**

Protein name: **Glycosidase crf1**

PFF score: **[66]**

Matched peptides No.: **[2]**  Sequence coverage %: **[11]** Matched sequences: **K.SISIVDYAGTDAPTTSSVR.E**

**R.EYLFGDHSGSWK.S**

Calculated Mr: **29591** Calculated *p*I: **4.47**

Probability Based Mowse Score:

Matched peptide sequences: shown in Bold Red:

Spot No.: **38**

NCBI accession No.: **gi|116010470**

Species: ***Gibberella fujikuroi***

Protein name: **FKBP-type peptidyl-prolyl cis-trans isomerase**

PFF score: **[64]**

Matched peptides No.: **[1]**  Sequence coverage %: **[12]** Matched sequences: **K.ALLDITPDYGYGAR.G**

Calculated Mr: **12048** Calculated *p*I: **4.85**

Probability Based Mowse Score:

Matched peptide sequences: shown in Bold Red:

Spot No.: **39**

NCBI accession No.: **gi|116010470**

Species: ***Gibberella fujikuroi***

Protein name: **FKBP-type peptidyl-prolyl cis-trans isomerase**

PFF score: **[101]**

Matched peptides No.: **[1]**  Sequence coverage %: **[12]** Matched sequences: **K.ALLDITPDYGYGAR.G**

Calculated Mr: **12048** Calculated *p*I: **4.85**

Probability Based Mowse Score:

Matched peptide sequences: shown in Bold Red:

Spot No.: **40**

NCBI accession No.: **gi|342877718**

Species: ***Fusarium oxysporum* Fo5176**

Protein name: **FKBP-type peptidyl-prolyl cis-trans isomerase**

PFF score: **[138]**

Matched peptides No.: **[3]**  Sequence coverage %: **[25]** Matched sequences: **K.GDQFDSSVGR.G**

**R.GAFVVPIGVGQVIK.G**

**K.ALLDITPDYGYGAR.G**

Calculated Mr:**16101**  Calculated *p*I: **9.30**

Probability Based Mowse Score:

Matched peptide sequences: shown in Bold Red:

Spot No.: **41**

NCBI accession No.: **gi|342875953**

Species: ***Fusarium oxysporum* Fo5176**

Protein name: **Glycosidase crf1**

PFF score: **[110]**

Matched peptides No.: **[2]**  Sequence coverage %: **[11]** Matched sequences: **K.SISIVDYAGTDAPTTSSVR.E**

**R.EYLFGDHSGSWK.S**

Calculated Mr: **29591** Calculated *p*I: **4.47**

Probability Based Mowse Score:

Matched peptide sequences: shown in Bold Red:

Spot No.: **42**

NCBI accession No.: **gi|342885951**

Species: ***Fusarium oxysporum* Fo5176**

Protein name: **Manganese superoxide dismutase precursor (sod-2)**

PFF score: **[173]**

Matched peptides No.: **[3]**  Sequence coverage %: **[17]** Matched sequences: **K.HHQAYVTNLNAALK.N**

**K.TWGSIQAFQEAFK.K**

**K.TLLGLQGSGWGWLVK.D**

Calculated Mr: **26531** Calculated *p*I: **6.22**

Probability Based Mowse Score:

Matched peptide sequences: shown in Bold Red:

Spot No.: **43**

NCBI accession No.: **gi|115395966**

Species: ***Aspergillus terreus* NIH2624**

Protein name: **60S acidic ribosomal protein P2**

PMF Mascot score:**76**  Sequence coverage %: **80**

Matched peptides No.:**7**  Total peptides No.:**26**

Calculated Mr: **11103** Calculated *p*I: **4.25**

Probability Based Mowse Score:

Matched peptide sequences: shown in Bold Red:

Spot No.: **44**

NCBI accession No.: **gi|342879607**

Species: ***Fusarium oxysporum* Fo5176**

PFF score: **[207]**

Protein name: **30 kDa heat shock protein**

Matched peptides No.: **[5]**  Sequence coverage %: **[33]** Matched sequences: **R.NFYNSDASFTPLFR.L**

**R.LLDDFDSYSR.Q**

**R.ETGEAYELHGELPGMTK.D + Oxidation (M)**

**K.DNVHIEFTEPQTMTIR.G + Oxidation (M)**

**K.AHESTEVTHHQQSK.E**

Calculated Mr: **24223** Calculated *p*I: **5.88**

Probability Based Mowse Score:

Matched peptide sequences: shown in Bold Red:

Spot No.: **45**

NCBI accession No.: **gi|302894417**

Species: ***Nectria haematococca* mpVI 77-13-4**

Protein name: **Isocitrate dehydrogenase (NAD)**

PMF Mascot score:**79**  Sequence coverage %: **39**

Matched peptides No.:**10** Total peptides No.:**49**

Calculated Mr: **41164** Calculated *p*I: **6.25**

Probability Based Mowse Score:

Matched peptide sequences: shown in Bold Red:

Spot No.: **46**

NCBI accession No.: **gi|342876330**

Species: ***Fusarium oxysporum* Fo5176**

Protein name: **Phosphoglycerate kinase**

PMF Mascot score:**175**  Sequence coverage %: **57**

Matched peptides No.:**22** Total peptides No.:**40**

Calculated Mr: **46156** Calculated *p*I: **6.31**

Probability Based Mowse Score:

Matched peptide sequences: shown in Bold Red:

Spot No.: **47**

NCBI accession No.: **gi|342890194**

Species: ***Fusarium oxysporum* Fo5176**

Protein name: **DEAD/DEAH box helicase**

PMF Mascot score:**126**  Sequence coverage %: **42**

Matched peptides No.:**20** Total peptides No.:**55**

Calculated Mr: **44984** Calculated *p*I: **4.98**

Probability Based Mowse Score:

Matched peptide sequences: shown in Bold Red:

Spot No.: **48**

NCBI accession No.: **gi|342887096**

Species: ***Fusarium oxysporum* Fo5176**

Protein name: **Pyruvate dehydrogenase (lipoamide) beta chain precursor (PDB1)**

PMF Mascot score:**123**  Sequence coverage %: **42**

Matched peptides No.:**15** Total peptides No.:**38**

Calculated Mr: **41784** Calculated *p*I: **5.72**

Probability Based Mowse Score:

Matched peptide sequences: shown in Bold Red:

Spot No.: **49**

NCBI accession No.: **gi|342882200**

Species: ***Fusarium oxysporum* Fo5176**

Protein name: **Beta-succinyl CoA synthetase precursor**

PFF score: **[135]**

Matched peptides No.: **[3]**  Sequence coverage %: **[9]** Matched sequences: **R.LCNAVYICER.K**

**K.ETPDAINTNYIDINVGVTDEVAR.E**

**K.FGFDDNAEFR.Q**

Calculated Mr: **48956** Calculated *p*I: **5.34**

Probability Based Mowse Score:

Matched peptide sequences: shown in Bold Red:

Spot No.: **50**

NCBI accession No.: **gi|342873590**

Species: ***Fusarium oxysporum* Fo5176**

Protein name: **Adenosylhomocysteinase**

PMF Mascot score:**93**  Sequence coverage %: **36**

Matched peptides No.:**19** Total peptides No.:**55**

Calculated Mr: **49506** Calculated *p*I: **5.57**

Probability Based Mowse Score:

Matched peptide sequences: shown in Bold Red:

Spot No.: **51**

NCBI accession No.: **gi|342877157**

Species: ***Fusarium oxysporum* Fo5176**

Protein name: **Pyruvate decarboxylase**

PMF Mascot score:**142**  Sequence coverage %: **52**

Matched peptides No.:**30** Total peptides No.: **56**

Calculated Mr: **63503** Calculated *p*I: **5.72**

Probability Based Mowse Score:

Matched peptide sequences: shown in Bold Red:

Spot No.: **52**

NCBI accession No.: **gi|336257873**

Species: ***Sordaria macrospora* k-hell**

Protein name: **Actin**

PMF Mascot score:**107**  Sequence coverage %: **67**

Matched peptides No.:**22** Total peptides No.:**84**

Calculated Mr: **38778** Calculated *p*I: **5.15**

Probability Based Mowse Score:

Matched peptide sequences: shown in Bold Red:

Spot No.: **53**

NCBI accession No.: **gi|342888181**

Species: ***Fusarium oxysporum* Fo5176**

Protein name: **Cytochrome-c oxidase chain IV precursor**

PFF score: **[66]**

Matched peptides No.: [2] Sequence coverage %: [18] Matched sequences: **R.TEEDLFGPGAAPGTVPTDLEQATGIER.L**

**K.TFADYIKPEYR.Y**

Calculated Mr: **22517** Calculated *p*I: **6.00**

Probability Based Mowse Score:

Matched peptide sequences: shown in Bold Red:

Spot No.: **54**

NCBI accession No.: **gi|342865956**

Species: ***Fusarium oxysporum* Fo5176**

Protein name: **Adenosine kinase**

PMF Mascot score:**118**  Sequence coverage %: **48**

Matched peptides No.:**12** Total peptides No.:**46**

Calculated Mr: **38124** Calculated *p*I: **5.42**

Probability Based Mowse Score:

Matched peptide sequences: shown in Bold Red:

Spot No.: **55**

NCBI accession No.: **gi|342885951**

Species: ***Fusarium oxysporum* Fo5176**

Protein name: **Manganese superoxide dismutase precursor (sod-2)**

PFF score: **[66]**

Matched peptides No.: **[2]** Sequence coverage %: **[12]** Matched sequences: **K.NYATATSTNDIAGQIALQSAIK.F**

**K.AAYVDNIWK.V**

Calculated Mr: **26531** Calculated *p*I:**6.22**

Probability Based Mowse Score:

Matched peptide sequences: shown in Bold Red:

Spot No.: **56**

NCBI accession No.: **gi|342885951**

Species: ***Fusarium oxysporum* Fo5176**

Protein name: **Manganese superoxide dismutase precursor (sod-2)**

PFF score: **[156]**

Matched peptides No.: **[2]** Sequence coverage %: **[12]** Matched sequences: **K.NYATATSTNDIAGQIALQSAIK.F**

**K.AAYVDNIWK.V**

Calculated Mr: **26531** Calculated *p*I: **6.22**

Probability Based Mowse Score:

Matched peptide sequences: shown in Bold Red:

Spot No.: **57**

NCBI accession No.: **gi|342876330**

Species: ***Fusarium oxysporum* Fo5176**

Protein name: **Phosphoglycerate kinase**

PFF score: **[258]**

Matched peptides No.: **[7]**  Sequence coverage %: **[24]** Matched sequences: **K.AEDGAVILLENLR.F**

**R.FHIEEEGSSK.D**

**K.GLTALGDVYINDAFGTAHR.A**

**R.AHSSMVGVDLPQK.A + Oxidation (M)**

**K.DGIPDGWQGLDCGEESVK.L**

**K.TILWNGPAGVFEFEK.F**

**K.LSHVSTGGGASLELLEGK.E**

Calculated Mr:46156 Calculated *p*I: 6.31

Probability Based Mowse Score:

Matched peptide sequences: shown in Bold Red:

Spot No.: **58**

NCBI accession No.: **gi|342888721**

Species: ***Fusarium oxysporum* Fo5176**

Protein name: **Members of the aldo/keto reductase family**

PFF score: **[272]**

Matched peptides No.: **[5]** Sequence coverage %: **[20]** Matched sequences: **K.REDIFITGK.L**

**K.TELDQGVTLSQTWEAVTK.L**

**K.DTGVTPAINQVER.H**

**R.SNFEEVELDEEAIK.E**

**K.TYSPDWDIDVFGDEK.E**

Calculated Mr: **37413** Calculated *p*I: **5.91**

Probability Based Mowse Score:

Matched peptide sequences: shown in Bold Red:

Spot No.: **59**

NCBI accession No.: **gi|342887616**

Species: ***Fusarium oxysporum* Fo5176**

Protein name: **Spermidine synthase**

PFF score: **[404]**

Matched peptides No.: **[6]**  Sequence coverage %: **[28]** Matched sequences: **R.EISDMWPGHAMTLR.V**

**K.STDFGNVLVLDNVIQCTER.D**

**K.VLVIGGGDGGVLR.E**

**K.HDCVEEATLCDIDEAVVR.L**

**K.SYFQLLHDALR.E**

**R.YYSAEIHK.A**

Calculated Mr: **33544** Calculated *p*I: **5.48**

Probability Based Mowse Score:

Matched peptide sequences: shown in Bold Red:

Spot No.: **60**

NCBI accession No.: **gi|342884543**

Species: ***Fusarium oxysporum* Fo5176**

Protein name: **Enolase**

PMF Mascot score:**115**  Sequence coverage %:**37**

Matched peptides No.:**14** Total peptides No.:**59**

Calculated Mr: **48508** Calculated *p*I: **5.17**

Probability Based Mowse Score:

Matched peptide sequences: shown in Bold Red:

Spot No.: **61**

NCBI accession No.: **gi|342877157**

Species: ***Fusarium oxysporum* Fo5176**

Protein name: **Pyruvate decarboxylase**

PMF Mascot score: **201**  Sequence coverage %: **52**

Matched peptides No.:**30** Total peptides No.:**73**

Calculated Mr: **63503** Calculated *p*I: **5.72**

Probability Based Mowse Score:

Matched peptide sequences: shown in Bold Red:

Spot No.: **62**

NCBI accession No.: **gi|342882246**

Species: ***Fusarium oxysporum* Fo5176**

Protein name: **Saccharopine dehydrogenase [NAD+, L-lysine-forming]**

PMF Mascot score:**176**  Sequence coverage %: **47**

Matched peptides No.:**22** Total peptides No.:**62**

Calculated Mr: **43568** Calculated *p*I: **5.18**

Probability Based Mowse Score:

Matched peptide sequences: shown in Bold Red:

Spot No.: **63**

NCBI accession No.: **gi|46111437**

Species: ***Gibberella zeae* PH-1**

Protein name: **Hypothetical protein**

PMF Mascot score: **128**  Sequence coverage %: **56**

Matched peptides No.:**15** Total peptides No.:**40**

Calculated Mr: **24829** Calculated *p*I: **5.20**

Probability Based Mowse Score:

Matched peptide sequences: shown in Bold Red:

Spot No.: **64**

NCBI accession No.: **gi|342879607**

Species: ***Fusarium oxysporum* Fo5176**

Protein name: **30 kDa heat shock protein**

PMF Mascot score:**191**  Sequence coverage %: **70**

Matched peptides No.:**20** Total peptides No.:**74**

Calculated Mr: **24223** Calculated *p*I: **5.88**

Probability Based Mowse Score:

Matched peptide sequences: shown in Bold Red:

Spot No.: **65**

NCBI accession No.: **gi|342881882**

Species: ***Fusarium oxysporum* Fo5176**

Protein name: **Glyceraldehyde-3-phosphate dehydrogenase**

PMF Mascot score:**100**  Sequence coverage %: **33**

Matched peptides No.:**13** Total peptides No.:**65**

Calculated Mr: **36213** Calculated *p*I: **6.11**

Probability Based Mowse Score:

Matched peptide sequences: shown in Bold Red:

Spot No.: **66**

NCBI accession No.: **gi|342871910**

Species: ***Fusarium oxysporum* Fo5176**

Protein name: **Fructose-bisphosphate aldolase**

PMF Mascot score:**212** Sequence coverage %: **61**

Matched peptides No.:**24** Total peptides No.:**75**

Calculated Mr: **37389** Calculated *p*I: **5.24**

Probability Based Mowse Score:

Matched peptide sequences: shown in Bold Red:

Spot No.: **67**

NCBI accession No.: **gi|46120358**

Species: ***Gibberella zeae* PH-1**

Protein name: **Mannitol dehydrogenase**

PFF score: **[368]**

Matched peptides No.: **[6]**  Sequence coverage %: **[38]** Matched sequences: **K.ANHILDLLSLK.G**

**K.VVVVTGASGPR.G**

**R.GAAEMGADVAITYASR.K**

**K.LNAADYNDVER.F**

**R.VNSISPGYIDTGLSDFIDAETQELWR.S**

**K.AAYVYFLSDASTYTTGSDLVIDGGYTCR.-**

Calculated Mr: **28590** Calculated *p*I: **5.58**

Probability Based Mowse Score:

Matched peptide sequences: shown in Bold Red:

Spot No.: **68**

NCBI accession No.: **gi|342884543**

Species: ***Fusarium oxysporum* Fo5176**

Protein name: **Enolase**

PMF Mascot score:**176**  Sequence coverage %: **57**

Matched peptides No.:**24** Total peptides No.:**61**

Calculated Mr: **48508** Calculated *p*I: **5.17**

Probability Based Mowse Score:

Matched peptide sequences: shown in Bold Red:

Spot No.: **69**

NCBI accession No.: **gi|342877157**

Species: ***Fusarium oxysporum* Fo5176**

Protein name: **Pyruvate decarboxylase**

PMF Mascot score:**142**  Sequence coverage %: **50**

Matched peptides No.:**22** Total peptides No.:**48**

Calculated Mr: **63503** Calculated *p*I: **5.72**

Probability Based Mowse Score:

Matched peptide sequences: shown in Bold Red:

Spot No.: **70**

NCBI accession No.: **gi|342877092**

Species: ***Fusarium oxysporum* Fo5176**

Protein name: **Hypothetical protein**

PMF Mascot score: **84**  Sequence coverage %: **35**

Matched peptides No.:**12** Total peptides No.:**72**

Calculated Mr: **24434** Calculated *p*I: **4.77**

Probability Based Mowse Score:

Matched peptide sequences: shown in Bold Red:

Spot No.: **71**

NCBI accession No.: **gi|342881882**

Species: ***Fusarium oxysporum* Fo5176**

Protein name: **Glyceraldehyde-3-phosphate dehydrogenase**

PMF Mascot score:**148**  Sequence coverage %: **44**

Matched peptides No.:**19** Total peptides No.: **54**

Calculated Mr: **36213** Calculated *p*I: **6.11**

Probability Based Mowse Score:

Matched peptide sequences: shown in Bold Red:

Spot No.: **72**

NCBI accession No.: **gi|342879240**

Species: ***Fusarium oxysporum* Fo5176**

Protein name: **Transaldolase**

PMF Mascot score:**168**  Sequence coverage %: **49**

Matched peptides No.:**20** Total peptides No.:**37**

Calculated Mr: **36821** Calculated *p*I: **5.29**

Probability Based Mowse Score:

Matched peptide sequences: shown in Bold Red:

Spot No.: **73**

NCBI accession No.: **gi|342884543**

Species: ***Fusarium oxysporum* Fo5176**

Protein name: **Enolase**

PFF score: **[129]**

Matched peptides No.: **[3]**  Sequence coverage %: **[11]** Matched sequences: **K.TQDIQIVGDDLTVTNPLR.I**

**R.SGETEDVTIADIAVGLR.A**

**R.IEEELGDQAIYPGANFR.K**

Calculated Mr: **48508** Calculated *p*I: **5.17**

Probability Based Mowse Score:

Matched peptide sequences: shown in Bold Red:

Spot No.: **74**

NCBI accession No.: **gi|342885916**

Species: ***Fusarium oxysporum* Fo5176**

Protein name: **TPI1-triose-phosphate isomerase**

PMF Mascot score:**149**  Sequence coverage %: **65**

Matched peptides No.:**16** Total peptides No.:**51**

Calculated Mr: **27245** Calculated *p*I: **5.29**

Probability Based Mowse Score:

Matched peptide sequences: shown in Bold Red:

Spot No.: **75**

NCBI accession No.: **gi|342881882**

Species: ***Fusarium oxysporum* Fo5176**

Protein name: **Glyceraldehyde-3-phosphate dehydrogenase**

PMF Mascot score:**162**  Sequence coverage %: **54**

Matched peptides No.:**19** Total peptides No.:**65**

Calculated Mr: **36213** Calculated *p*I: **6.11**

Probability Based Mowse Score:

Matched peptide sequences: shown in Bold Red:

Spot No.: **76**

NCBI accession No.: **gi|302925302**

Species: ***Nectria haematococca* mpVI 77-13-4**

Protein name: **Enolase**

PFF score: **[607]**

Matched peptides No.: **[6]**  Sequence coverage %: **[19]** Matched sequences: **R.GNPTVEVDVVTETGLHR.A**

**R.AIVPSGASTGQHEACELR.D**

**K.TQDIQIVGDDLTVTNPLR.I**

**K.DSYADGWGVMVSHR.S**

**K.DSYADGWGVMVSHR.S + Oxidation (M)**

**R.SGETEDVTIADIAVGLR.A**

Calculated Mr: **47231** Calculated *p*I: **4.92**

Probability Based Mowse Score:

Matched peptide sequences: shown in Bold Red:

Spot No.: **77**

NCBI accession No.: **gi|342883857**

Species: ***Fusarium oxysporum* Fo5176**

Protein name: **Hsp70-like protein**

PFF score: **[446]**

Matched peptides No.: **[8]**  Sequence coverage %: **[14]** Matched sequences: **R.TTPSVVAFAEDGER.L**

**K.IVQHTNGDAWVAAR.G**

**R.GQNYSPSQIGGFVLNK.M**

**K.NAVVTVPAYFNDSQR.Q**

**K.DAGQIAGLNVLR.V**

**K.EIQEVILVGGMTR.M**

**K.EIQEVILVGGMTR.M + Oxidation (M)**

**K.LLGNFQLVGIPPAHR.G**

Calculated Mr: **73180** Calculated *p*I: **5.68**

Probability Based Mowse Score:

Matched peptide sequences: shown in Bold Red:
